# COVID-19 mRNA vaccines drive differential Fc-functional profiles in pregnant, lactating, and non-pregnant women

**DOI:** 10.1126/scitranslmed.abi8631

**Published:** 2021-10-27

**Authors:** Caroline Atyeo, Elizabeth A. DeRiso, Christine Davis, Evan A. Bordt, Rose M. De Guzman, Lydia L. Shook, Lael M. Yonker, Alessio Fasano, Babatunde Akinwunmi, Douglas A. Lauffenburger, Michal A. Elovitz, Kathryn J. Gray, Andrea G. Edlow, Galit Alter

**Affiliations:** 1. Ragon Institute of MGH, MIT, and Harvard, Cambridge, MA 02139, USA.; 2. PhD Program in Virology, Division of Medical Sciences, Harvard University, Boston, MA 02115, USA.; 3. Department of Biological Engineering, Massachusetts Institute of Technology, Cambridge, MA 02139, USA.; 4. Department of Pediatrics, Lurie Center for Autism, Massachusetts General Hospital, Harvard Medical School, Boston, MA, 02114, USA.; 5. Department of Obstetrics and Gynecology, Massachusetts General Hospital, Harvard Medical School, Boston, MA 02114, USA.; 6. Vincent Center for Reproductive Biology, Massachusetts General Hospital, Boston, MA, 02114, USA.; 7. Mucosal Immunology and Biology Research Center, Massachusetts General Hospital, Boston, MA; Department of Pediatrics, Massachusetts General Hospital, Boston, MA; Harvard Medical School, Boston, MA, 02115, USA.; 8. Department of Obstetrics and Gynecology, Brigham and Women’s Hospital, Harvard Medical School, Boston, MA, 02115, USA.; 9. Maternal and Child Health Research Center, Perelman School of Medicine, University of Pennsylvania, Philadelphia, PA, 19104, USA.

## Abstract

Substantial immunological changes occur throughout pregnancy to promote tolerization of the mother to the fetus and allow fetal growth. However, additional local and systemic immunological adaptations also occur, allowing the maternal immune system to continue to protect the dyad against pathogens both during pregnancy and after birth through lactation. This fine balance of tolerance and immunity, along with physiological and hormonal changes, contribute to increased susceptibility to particular infections in pregnancy, including more severe coronavirus disease 2019 (COVID-19). Whether these changes also make pregnant women less responsive to vaccination or induce altered immune responses to vaccination remains incompletely understood. To holistically define potential changes in vaccine response during pregnancy and lactation, we deeply profiled the humoral vaccine response in a group of pregnant and lactating women and non-pregnant age-matched controls. Vaccine-specific titers were comparable between pregnant women, lactating women, and non-pregnant controls. However, Fc receptor (FcR)-binding and antibody effector functions were induced with delayed kinetics in both pregnant and lactating women compared to non-pregnant women after the first vaccine dose, which normalized after the second dose. Antibody boosting resulted in high FcR-binding titers in breastmilk. These data suggest that pregnancy promotes resistance to generating highly inflammatory antibodies and indicates that there is a critical need to follow prime-boost timelines in this vulnerable population to ensure full immunity is attained.

## One Sentence Summary

Pregnant and lactating women develop distinct antibody Fc profiles in response to the mRNA-1273 and BNT162b2 vaccines compared to non-pregnant women.

## Introduction:

Pregnant women experience both increased disease severity and morbidity upon severe acute respiratory syndrome coronavirus 2 (SARS-CoV-2) infection (1, 2). However, pregnant and lactating women were left out of initial coronavirus disease 2019 (COVID-19) vaccine trials due to heightened safety concerns (3–6). Given that pregnant women are vulnerable to severe COVID-19, it is important to understand the immunological response to vaccination in pregnant and lactating women. Understanding how pregnancy and lactation affect responses to vaccination and antibody transfer to infants offers critical opportunities to guide recommendations for this population.

Pregnant and lactating women are routinely encouraged to receive vaccines against influenza and pertussis (7, 8). Mounting data point to dampened vaccine-induced antibody responses in pregnant women marked by a lower fold increase in antibody titers, lower neutralizing antibody responses, and reduced T cell immune responses compared to non-pregnant women (9). Given the substantial morbidity associated with influenza infection in pregnant women annually, influenza vaccination is recommended throughout gestation (10), whereas pertussis vaccination is recommended in the late second and early third trimester to facilitate maximal antibody transfer and protect the developing neonate (11–13). However, because most women have been exposed or previously immunized against these pathogens, these vaccines largely boost immunity, rather than prime a de novo immune response. Thus, whether the same principles for antibody transfer will apply to additional vaccine platforms used against SARS-CoV-2, as well as to a new antigen (SARS-CoV-2 spike protein), remains unclear. Moreover, whether vaccine-induced immune profiles will vary across pregnancy and lactation, impacting antibody transit across the placenta or into breastmilk, is not known. This understanding could provide crucial insights to guide the optimal administration of the vaccine to women and their infants.

The first two vaccines that were approved for emergency use authorization (EUA) by the Food and Drug Administration (FDA) use mRNA to induce an immune response against the SARS-CoV-2 spike protein. Linked to highly effective protection against severe COVID-19 disease in non-pregnant populations (14, 15), both mRNA platforms clearly lead to the induction of robust immunity in men and non-pregnant women across age groups (16, 17). Moreover, emerging data suggest that mRNA vaccines also induce comparable antibody titers and neutralization in pregnant and lactating women, linked to transfer of antibodies to neonates (18, 19). The development of multiple vaccine platforms, coupled with the availability of high-dimensional antibody profiling technologies, enables the unprecedented opportunity to dissect the de novo mRNA vaccine-induced immune response in this vulnerable population.

In addition to the role of antibodies in binding and neutralization, antibodies contribute to protection against COVID-19 disease through their ability to recruit the innate immune response with their Fc-domain (20, 21). The Fc-functions of the humoral immune response play a critical role in resolution of COVID-19 (22), are associated with protection from infection following vaccination (23), play a critical role in antibody transfer across the placenta (24–27) and may also influence transfer into breastmilk (28). Although previous studies have shown that mRNA vaccines are immunogenic in pregnant and lactating women (29, 30), no studies have characterized the Fc-profile of mRNA vaccine-induced antibodies in pregnant and lactating women. To define qualitative features of the vaccine-induced humoral immune response across pregnancy and early life, we comprehensively profiled the humoral immune response following mRNA vaccination in pregnant, lactating, or non-pregnant women who received either the BNT162b2 or mRNA-1273 vaccines. Our data point to differences in vaccine-induced antibody profiles among pregnant, lactating, and non-pregnant women that influence the abundance and quality of antibodies to neonates, arguing for a need to understand how timing of vaccine administration in pregnancy impacts both maternal immune response and antibody transfer to neonates.

## Results

### Vaccination induces distinct antibody responses in pregnant, lactating, and non-pregnant women.

Two mRNA vaccines were the first EUA-approved vaccines, showing about 95% protection against severe COVID-19 (14, 31–33). Emerging data have begun to illustrate the robust immunogenicity of these vaccines in pregnant and lactating women, in the absence of enhanced reactogenicity (18). However, whether the overall humoral immune profile diverges in pregnant or lactating women and if these profiles impact transfer to neonates remains incompletely understood. Therefore, we characterized the SARS-CoV-2 humoral immune response in a cohort of 84 pregnant, 31 lactating and 16 non-pregnant age-matched controls vaccinated with BNT162b2 or mRNA-1273 and whose antibody isotype response was previously studied in our lab (29). Individuals were sampled after first vaccination (post-prime, at the time of second dose) or after second vaccination (post-boost, 2–5.5 weeks following 2^nd^ dose) (Table 1). A subset of individuals was sampled after both time points.

**Figure 1.**
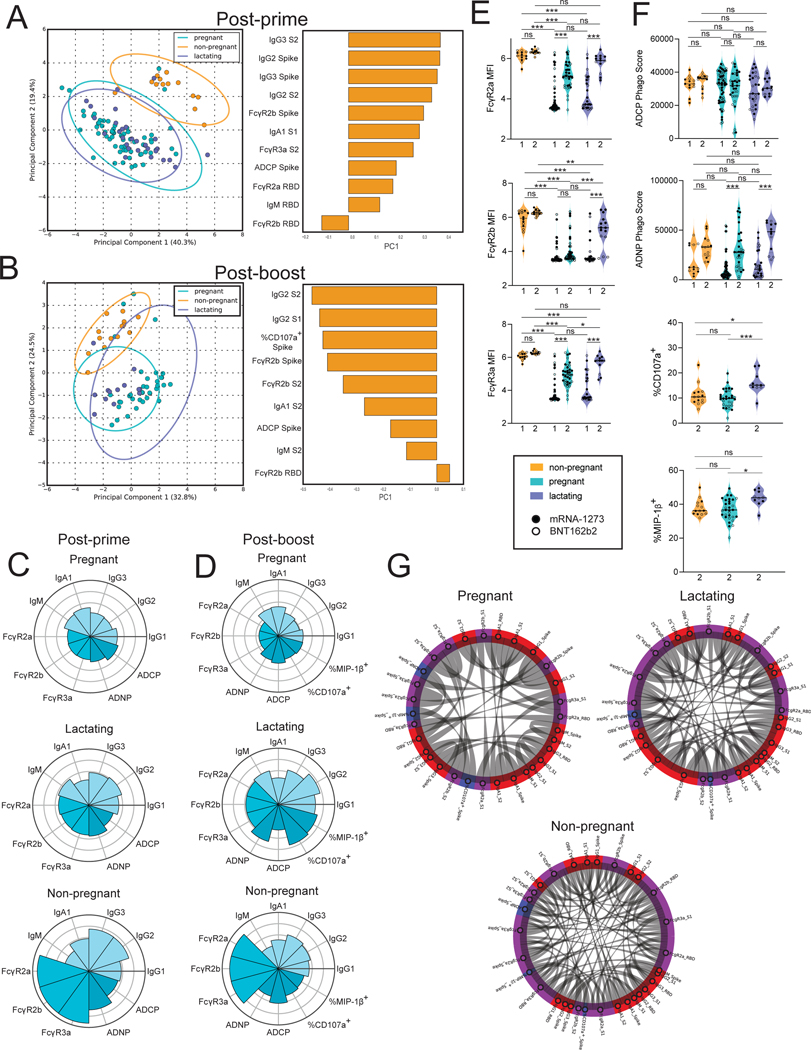
Vaccination induces enhanced FcR-binding in non-pregnant women. **A and B.** A principal component analysis (PCA) was built using LASSO-selected antibody features at 3 to 4 weeks post prime vaccination (A) or 2 to 5.5 weeks post boost vaccination (B). The dot plots show the scores of each individual, with each dot representing an individual. The ellipses represent the 95% confidence interval for each group. The bar plots show the loadings of the LASSO-selected features along principal component 1 (PC1). **C and D**. The polar plots show the mean percentile rank for each feature for post-prime (C) and post-boost (D) samples. Features were ranked separately for each time point. **E**. The violin plots show the FcγR-binding for non-pregnant, pregnant, and lactating women at post-prime (1) (non-pregnant n = 13, pregnant n = 64, lactating n = 28) and post-boost (2) (non-pregnant n = 14, pregnant n = 36, lactating n = 13). The filled dots show the titer for women who received mRNA-1273, and outlines show the titer for women who received BNT162b2. MFI, median fluorescence intensity. Data are presented as median ± IQR. Significance was determined by a one-way ANOVA followed by posthoc Tukey’s multiple comparison test. P-values were then corrected for multiple comparisons using the Bejamini-Hochberg procedure, * p <0.05, ** p < 0.01,*** p < 0.001, **** p < 0.0001, ns, not significant. **F**. The violin plots show the antibody functions for non-pregnant, pregnant, and lactating women at post-prime (1) (non-pregnant n = 13, pregnant n = 64, lactating n = 28) and post-boost (2) (non-pregnant n = 14, pregnant n = 36, lactating n = 13). The filled dots show the titer for women who received mRNA-1273, and outlines show the titer for women who received BNT162b2 vaccine. Phago indicates phagocytosis. Data are presented as median ± IQR. Significance was determined by a one-way ANOVA followed by posthoc Tukey’s multiple comparison test. P-values were then corrected for multiple comparisons using the Bejamini-Hochberg procedure, * p <0.05, ** p < 0.01,*** p < 0.001, **** p < 0.0001, ns, not significant. **G**. The chord diagrams connect the features that have a spearman correlation > 0.75 and Bonferroni-corrected p-value < 0.05 for non-pregnant, pregnant, and lactating women. Red indicates antibody isotype, purple indicates FcR-binding and blue indicates antibody function.

To visualize the differences in the Fc-profiles across the three populations, we performed unsupervised principal component analysis (PCA). At the post-prime timepoint (3 to 4 weeks post first immunization), clear differences were noted between serum antibody responses of pregnant/lactating women and non-pregnant women (Fig. 1A and fig. S1A). Most differences observed in samples collected after the first vaccine dose related to lower antibody titers (principal component 1, PC1) and FcR-binding capacity (principal component 2, PC2) among pregnant and lactating women compared with non-pregnant women. In samples collected 2 to 5.5 weeks after boost vaccination (post-boost), there was a decrease in the separation between pregnant or lactating and non-pregnant women (Fig. 1B and fig. S1B). Although some differences persisted, differences were nearly exclusively linked to enhanced FcR-binding in non-pregnant women.

To further understand the difference in individual antibody features in pregnant, lactating, and non-pregnant women, we plotted the mean percentile rank of each spike protein-specific feature measured at the post-prime and post-boost timepoints (Fig. 1C and D). At the post-prime timepoint, non-pregnant women had higher IgG subclass responses, higher antibody functions and higher FcR-binding compared to pregnant and lactating women (Fig. 1C). At this time point, pregnant and lactating women had similar antibody responses. Interestingly, in samples collected after boost vaccination, lactating women boosted their antibody response more effectively than pregnant women, marked by higher IgG titers (Fig. 1D). In addition, lactating women displayed higher natural killer (NK) cell activity than pregnant women, based on the percent of CD107a^+^ cells, a degranulation marker, and the percent of Macrophage inflammatory protein (MIP)-1β+ cells, a chemokine that is produced by activated NK cells. This suggests that lactating women make qualitatively different responses to the second dose of vaccine compared to pregnant individuals (Fig. 1D). After receiving a booster vaccine dose, the vaccine response in lactating women was similar to that of non-pregnant women, although lactating women had lower FcR-binding compared to non-pregnant women.

Next, to specifically capture the differences in the quality of the vaccine-induced humoral immune response across the participants, isotype, FcR-binding, and Fc-effector profiles were compared in a univariate manner across the groups. Previously, our group has shown that there were no differences noted in IgG1, IgA, and IgM antibody production across the groups (29). In contrast, we observed that FcR-binding antibodies across all FcRs were higher in non-pregnant compared to pregnant and lactating women post-prime vaccination (Fig. 1E and fig. S1C). However, in samples collected post-boost vaccination, both pregnant and lactating women raised FcR-binding antibodies, although all FcR-binding antibodies remained lower in pregnant women compared to non-pregnant, whereas FcγR2b-binding antibodies remained lower in lactating women after a second vaccine dose (Fig. 1E). All three populations induced similar antibody-dependent cellular phagocytosis (ADCP) and did not increase ADCP function post-boost vaccination. In contrast antibody-dependent neutrophil phagocytosis (ADNP) activity was increased in pregnant and lactating women after boosting (Fig. 1F and fig. S1D). The ability of antibodies to drive NK cell activation was distinct across the groups, and lactating women induced higher NK cell activating antibodies (based on CD107a^+^ expression) after receiving a booster vaccine compared to pregnant women and non-pregnant women (Fig. 1F). Antibodies isolated from lactating women also induced a greater percentage of MIP-1β^+^ NK cells than antibodies from pregnant women (Fig. 1F). These higher FcR-binding profiles in non-pregnant and lactating women were linked to enhanced coordination in the humoral immune responses compared to pregnant women, the latter showing sparser coordination in the vaccine induced humoral immune response (Fig. 1G). Overall, these data point to deficiencies in the ability of pregnant women to generate functional, but not total, antibodies with boosting compared to lactating women. Further, these data suggest that pregnant and lactating women show potential early alterations in vaccine-induced immune responses that improve after a booster vaccine.

### Antibody profiles differ between maternal serum and cord blood.

**Figure 2.**
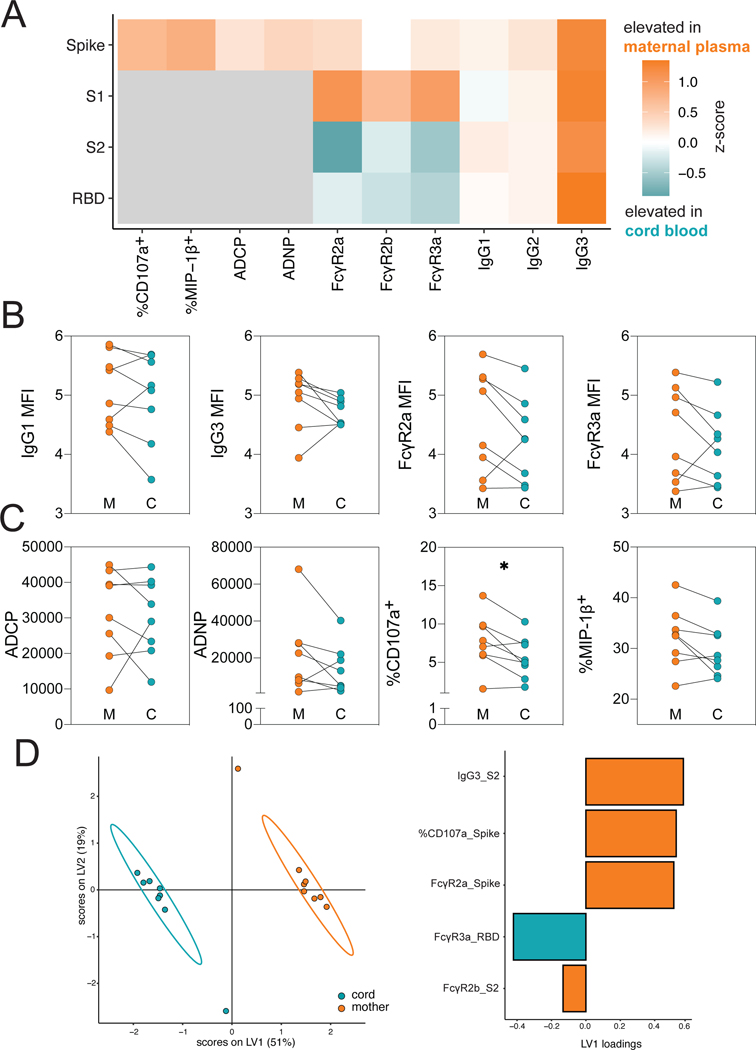
Vaccination results in the transfer of FcR-binding antibodies into the cord blood. **A**. The heatmap shows the difference in median z-score between maternal plasma and cord blood (n = 8 for both maternal and cord) for each SARS-CoV-2-specific feature. Orange indicates the feature was elevated in maternal plasma, and teal indicates that the features was elevated in cord blood. Gray boxes indicate that the experiment was not performed. Significance was measured by a Mann-Whitney U test and p-values were Benjamini-Hochberg adjusted. No significant differences were observed. **B**. The dot plots show the IgG1, IgG3, FcγR2a-binding and FcγR3a-binding titer against spike protein for maternal (M) and cord (C) blood. Lines connect maternal:cord dyads (n = 8). Significance was determined by Wilcoxon-matched pairs signed rank test. **C**. The dot plots show the ADCP, ADNP and ADNKA (CD107a^+^ or MIP-1β+) functional titers against spike protein for maternal (M) and cord (C) blood. Lines connect maternal:cord dyads (n= 8). * p <0.05. **D**. A multilevel partial least-squares discriminant analysis (mPLSDA) was built using maternal or cord LASSO-selected SARS-CoV-2-specific features. The dot plot (left) shows the scores of each sample, with the ellipses representing the 95% confidence interval for each group (n = 8 for both groups). The bar plot (right) shows the loadings of each LASSO-selected feature for the mPLSDA. The color of the bar indicates in which group the feature is enriched.

Previous studies focused on pertussis vaccination have pointed to the selective and active transfer of highly functional antibodies across the placenta, marked by the specific selective transfer of FcγR3a binding antibodies (25). However, more recent studies of SARS-CoV-2 infection in pregnancy have noted compromised transfer with infection in the third trimester, linked to reduced antibody transfer (transfer ratio < 1) but maintaining a selection bias based on binding to FcγR3a (27). To begin to understand the overall profiles of antibodies that are transferred from pregnant individuals to infants, we profiled the humoral immune responses across 8 maternal:umbilical cord blood dyads. The median days from prime to delivery in these 8 women was 37 days (25.75–41.5). Overall, higher titer of antibodies were observed in maternal blood compared to cord blood (Fig. 2A). Variable patterns of transfer of IgG titer, FcR-binding and antibody function were observed from the mother to the cord (Fig. 2B and C). Despite the recency of vaccination, equivalent, IgG1 spike protein-specific titers were transferred across the placenta to the infant (Fig. 2B). Despite previous observations of augmented NK cell activating antibody transfer following vaccines that boost previously established immunity, such as to pertussis and influenza (25), stable phagocytic antibodies but decreased NK-cell activating antibodies (*P* = 0.039, %CD107a^+^) were transferred to infants (Fig. 2C). Conversely, no loss of transfer of NK cell activating antibodies was noted for influenza hemagglutinin (HA)-specific antibodies in the same mother:cord pairs (fig. S2A and B) suggesting that reduced transfer of spike protein-specific antibodies may not be attributable to vaccine-induced changes in placental activity. Instead, decreased spike protein-specific transfer could be linked to time from vaccination (fig. S2C and D), suggesting that vaccination proximal to the time of birth may simply not permit the effective transfer of the most functional antibody subpopulations.

We next aimed to determine whether placenta transfer was strictly governed by total amounts of antibody or based on specific characteristics of the vaccine-induced humoral immune response. Thus, we performed a multilevel partial least squares discriminant analysis (mPLSDA) to determine the spike protein-specific antibody features that transferred preferentially across the cord (Fig. 2D). This supervised dimensionality-reduction method aims to identify the minimal matched-features that best discriminate between mother and cord samples. Given the highly correlated nature of the humoral immune response, the least absolute shrinkage and selection operator (LASSO) first aims to conservatively reduce the number of features to the minimal number of features able to most effectively discriminate between the maternal:cord samples to prevent overfitting. Fc-profiles in maternal and cord blood were completely distinct, with expected higher overall titers of antibodies in maternal blood, as the vaccine was only administered in these dyads in the third trimester. However, despite the lower titers of antibodies in the cord, receptor binding domain-specific FcγR3a binding was enriched in the cord (Fig. 2D, right). Thus, similar to the previously observed transfer sieve, even at low antibody-transfer rates, the placenta selects for FcγR3a-binding, functionally enhanced vaccine-induced antibodies.

### Optimal vaccine-induced antibody transfer to the breastmilk requires boosting

**Figure 3.**
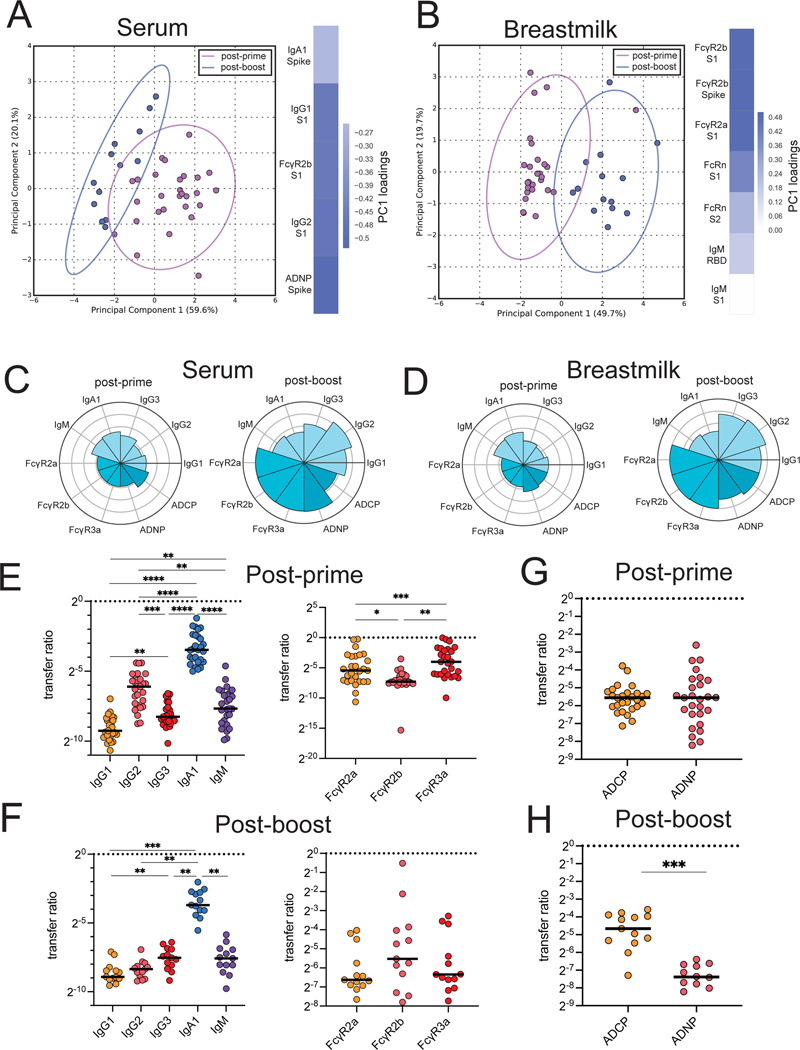
Second dose of vaccination is required for boost in functional antibody response in serum and breastmilk of lactating women. **A.** A PCA was built on LASSO-selected SARS-CoV-2-specific features from samples collected post-prime vaccination (n = 28) (purple) and from samples collected post-boost vaccination (n = 13) (blue) in lactating women. The ellipses represent the 95% confidence interval for each group. The heatmaps show the contribution of each feature along each principal component (PC). The color of the heatmap indicates in which group each feature is enriched. Blue indicates that all features were enriched post-boost. **B**. A PCA was built on LASSO-selected SARS-CoV-2-specific features in breastmilk samples collected post-prime vaccination (purple) or post-boost vaccination (blue), using timepoint as the outcome variable. The ellipses represent the 95% confidence interval for each timepoint. The heatmap shows the contribution of each feature to the corresponding PC. **C**. The polar plots show the mean percentile rank for each feature for post-prime or post-boost samples in the serum of lactating women. **D**. The polar plots show the mean percentile rank for each feature for post-prime or post-boost samples in breastmilk. **E**. The dot plot shows the transfer ratio of breastmilk:serum for each isotype (left) and FcR (right) measured 3 to four weeks post-prime vaccination. Horizontal dotted lines indicate a transfer ratio of 1. Horizontal bars indicate median. Significance was determined by a one-way ANOVA followed by Tukey’s multiple comparison correction. * p <0.05, ** p < 0.01, *** p < 0.001, **** p < 0.0001. **F**. The dot plot shows the transfer ratio of breastmilk:serum for each isotype (left) and FcR (right) measured 2 to 5.5 weeks post-boost vaccination. Horizontal dotted lines indicate a transfer ratio of 1. Horizontal bars indicate median. Significance was determined by a one-way ANOVA followed by Tukey’s multiple comparison correction. ** p < 0.01,*** p < 0.001. **G**. The dot plots show the transfer ratio of breastmilk:serum between ADCP and ADNP measured post-prime vaccination. The horizontal dotted line indicates a transfer ratio of 1. Horizontal bars indicate median. Significance was determined by Wilcoxon-matched pairs signed rank test. No significant difference was observed. **H**. The dot plots show the transfer ratio of breastmilk:serum between ADCP and ADNP measured post-boost vaccination. The horizontal dotted line indicates a transfer ratio of 1. Horizontal bars indicate median. Significance was determined by Wilcoxon-matched pairs signed rank test. p < 0.001.

Beyond placental transfer, antibody transfer can continue to occur after birth through breastmilk. Vaccine immunity changes over time in immunized lactating women, but how this influences antibody transfer to the infant is incompletely understood. Using an unsupervised PCA of the post-prime and post-boost vaccine-induced immune responses, we observed a slightly expanded antibody functional and FcR-binding response in the serum of lactating women after receiving a booster vaccine dose (Fig. 3A and fig. S3), suggesting a functional maturation of the humoral immune response with boosting. Similarly, in breastmilk, boosting resulted in increased transfer of FcR-binding antibodies and functional antibodies (Fig. 3B and fig. S3, S4A). To gain specific insights into the antibody subpopulations that are transferred most efficiently across the blood and breastmilk, we next plotted the mean percentile rank of each spike protein-specific antibody feature at post-prime and post-boost (Fig. 3C and D). The polar plots highlight the preferential boosting of FcR-binding IgG responses in the serum after a booster vaccine dose, with a prominent expansion of IgG and FcR binding, with no obvious impact to IgA and IgM responses (Fig. 3C). Moreover, the same transfer profile was noted in the breastmilk (Fig. 3D), with a high transfer of IgG antibodies with FcR-binding capabilities 2 to 5.5 weeks post booster vaccination. To further understand the sieve of antibodies from serum to breastmilk upon vaccination, we plotted the transfer ratio (breastmilk median fluorescence intensity (MFI):serum MFI) of isotypes and FcRs against the SARS-CoV-2 spike protein at the post-prime timepoint (Fig. 3E) and at the post-boost timepoint (Fig. 3F and fig. S4B). At both post-prime and post-boost, IgA was the most preferentially transferred of any isotype. Interestingly, IgG2 was transferred more highly to breastmilk than any other IgG subclass at the post-prime timepoint, but at the post-boost timepoint, IgG3 was the most highly transferred subclass. Moreover, at the post-prime timepoint, there was a preferential transfer of antibodies that could bind FcγR3a, whereas antibodies that could bind FcγR2b, the inhibitory FcR, had the lowest transfer ratio. At the post-boost timepoint, however, there were no differences in the transfer of FcR-binding antibodies, pointing to robust transfer of all FcR-binding antibodies to breastmilk after vaccine boost. In addition, we analyzed the transfer ratio of antibody functions at the post-prime and post-boost timepoints. Whereas ADCP and ADNP were transferred at equivalent ratios at the post-prime timepoint (Fig. 3G), antibodies able to drive ADCP were transferred at a higher ratio than those able to activate ADNP after boost (Fig. 3H), likely reflecting the enhanced ADNP activity in the serum of lactating women after boost. In addition, NK-cell activating antibodies had a low transfer ratio at the post-boost timepoint (fig. S4C), suggesting a sieve at the mammary gland, preventing the transfer of highly inflammatory antibodies through breastmilk. This analysis revealed enhanced functional antibodies in breastmilk following the boost, accompanied by decreased IgM and IgA induction post-boost. Collectively, these data emphasize that, although the breast clearly enriches IgM and IgA delivery to the breastmilk (34, 35),vaccination appears to augment highly functional IgG transit to the milk that are likely key to antiviral immunity across viral pathogens (35).

### mRNA-1273 and BNT162b2 vaccination induce differential antibody responses in pregnant and lactating women.

**Figure 4.**
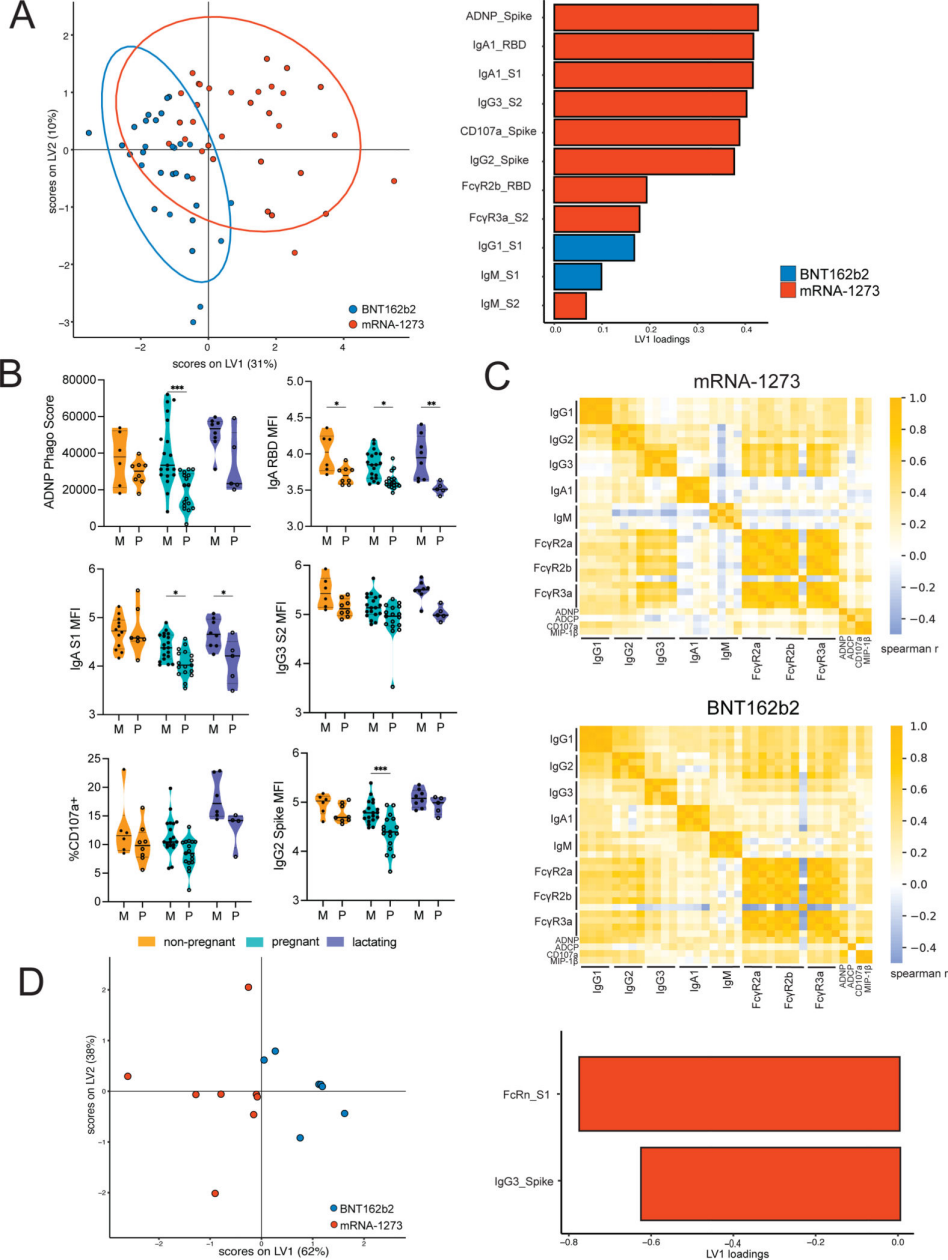
The antibody response elicited by mRNA-1273 and BNT162b2 vaccination differ in pregnant and lactating women. A. A LASSO-PLSDA model was built on post-boost vaccination data from all groups. Samples were collected 2 to 5.5 weeks post booster administration. The dot plot (left) shows the scores for each sample, with each dot representing a sample. The ellipses represent the 95% confidence interval for each group. The bar plot (right) shows the loadings of each LASSO-selected feature, where the color marks the group enrichment. B. The violin plots show differences in the top LASSO-selected features from (A) in non-pregnant (n = 8 for BNT162b2, n = 6 for mRNA-1273), pregnant (n = 17 for BNT162b2, n = 19 for mRNA-1273), and lactating (n = 5 for BNT162b2, n = 8 for mRNA-1273) women given either mRNA-1273 (M, filled dots) or BNT162b2 (P, outline dots). All data in the violin plots is from samples collected 2 to 5.5 weeks post second vaccine dose. Significance was determined by a one-way ANOVA followed by posthoc Šidák’s multiple comparison test. P-values were then corrected for multiple comparisons using the Bejamini-Hochberg procedure, * p <0.05, ** p < 0.01,*** p < 0.001. C. The heatmaps show the spearman correlation between features for women who received the mRNA-1273 (top) or BNT162b2 (bottom). For isotypes and FcγR-binding, the features are ordered as spike protein, S2, S1, and RBD. For functional data, only functional activity against spike protein was measured. D. A LASSO-PLSDA model was built on post-booster vaccine breastmilk data. The dot plot (left) shows the scores for each sample, with each dot representing a sample (n = 6 for BNT162b2, n = 6 for mRNA-1273). The ellipses represent the 95% confidence interval for each group. The bar plot (right) shows the loadings of each LASSO-selected feature, where the color marks the group enrichment.

Although both mRNA-1273 and BNT162b2 exploit mRNA-based technologies, differences in mRNA dosage, lipid carriers, and vaccine dosing regimens may alter the quality of the humoral immune response across the vaccines. Thus, we compared immune responses across all women dosed with these mRNA vaccines. To do this, a partial least squares discriminant analysis (PLSDA) was performed, aimed at determining whether the vaccine-induced humoral profiles induced by these vaccines differ and to define the specific humoral features that diverge across the vaccines. Although no differences were noted across the vaccine-induced immune responses in samples collected post-prime vaccination (fig. S5A), separation was observable in the vaccine-induced antibody profiles in samples collected post-boost vaccination (Fig. 4A). Notably,mRNA-1273-vaccinated women exhibited enriched neutrophil activating antibodies (ADNP), higher titers of vaccine-specific IgA, IgG2, IgG3, and NK cell activating antibodies (CD107a^+^) compared to BNT162b2 vaccinated women (Fig. 4A, fig. S5B to D). Conversely, women receiving BNT162b2 exhibited a slight enrichment of IgG1 and FcγR3A-binding (Fig. 4A) humoral immune responses. To further dissect these differences at the post-boost timepoint, women were split into groups by their pregnancy and lactation status. Although differences in receptor binding domain (RBD)-specific IgA1 titers were noted across all groups, differences in ADNP were only amplified in pregnant women (Fig. 4B).

Moreover, to further understand the functional basis for these differences, we examined the functional coordination of the humoral immune response induced by each vaccine across the three populations of women (Fig. 4C). We observed that mRNA-1273 vaccination resulted in a more focused coordination in the humoral immune response, centered around a high IgG1/IgG3 response with robust FcR-binding and functional coordination. Conversely, women receiving BNT162b2 generated a broader coordinated immune response including IgG2 and IgM responses and the exclusion of monocyte phagocytosis (ADCP), potentially suggesting a more diffuse overall humoral immune coordination profile. These serum differences translated to differences in antibodies transferred in breastmilk (Fig. 4D), with enhanced FcR-binding antibody and functional IgG3 antibody transfer to breastmilk observed in mRNA-1273-immunized lactating women. Whether these differences are attributable to dose, lipids, or timing of vaccination remains unclear, but provide clues that mRNA platforms may be selectively deployed to enhance protection in neonates once precise mechanistic correlates of immunity are defined.

## Discussion

Both EUA-approved COVID-19 mRNA vaccines have been shown to be safe and highly immunogenic in non-pregnant populations (14, 15), and emerging data suggest that the vaccines are immunogenic and similarly reactogenic in pregnant and lactating women (18). However, pregnancy and lactation represent distinct immunological states (36, 37), that have been previously associated with reduced immunogenicity (9). Whether this unique immune state is associated with the evolution of distinct humoral immune profiles upon vaccination remains incompletely understood. Using systems serology, we observed changes in the magnitude, kinetics, and quality of functional profiles of vaccine-induced antibodies. We additionally found differences in the overall antibody profile across women receiving mRNA-1273 and BNT162b2. These findings collectively point to an extended window of vulnerability in pregnancy and lactation following vaccination, requiring timely boosting to achieve fully functional matured antibodies to protect the pregnant individual and their offspring.

Pregnancy represents a delicate immunological balance which has been associated with enhanced vulnerabilities to infection in pregnant women who experience more severe influenza infection and SARS-CoV-2 infection (38, 39). This vulnerability has been linked to dampened, rather than blocked, pro-inflammatory immunity, with reduced responsiveness to vaccination (9). Beyond quantitative measures of pregnancy-associated changes, less is known about the qualitative functional changes in the vaccine-induced humoral response during pregnancy. Here, we observed a delay in the evolution of FcR-binding and functional antibody responses in pregnant and lactating women after initial vaccination against a de novo pathogen. Conversely, we observed higher functional antibodies for NK cell activity and neutrophil phagocytosis in lactating women compared to both pregnant and non-pregnant women upon boost. These data point to distinct response profiles across each of these immunological states, raising the possibility that vaccines may drive different antibody functional profiles, programmed evolutionarily to maximize protection for the mother-baby dyad in that unique immune state. Given the low responsiveness to vaccination after prime, these data also highlight the critical importance to adhere to vaccination boosting among this population to optimize immunity in pregnant and lactating women. In addition, the pregnant women in this study were immunized across all trimesters of pregnancy. Given the immunological changes that occur throughout the course of pregnancy (40, 41), further studies should aim to understand how the overall kinetics (post-prime and post-boost), quality, and magnitude of immune responsiveness to vaccination varies over gestation.

Vaccination during pregnancy increases the passive protection transferred to newborns, who are at increased risk for severe disease upon infection due to their immature immune system. Although few newborns have been infected with SARS-CoV-2, those who have been infected have more severe outcomes than older children (42–44). In the case of natural infection, poor placental transfer of antibodies has been observed in women infected in the third trimester, particularly pronounced in mothers carrying a male fetus, but these transfer ratios increase to an expected transfer ratio above 1 in women infected earlier in pregnancy (45–47). Likewise, women in our study that gave birth were all immunized in the third trimester and had transfer ratios below one, likely reflective of the proximity of vaccination to delivery. In accordance with this, a previous study has shown that the transfer of SARS-CoV-2-specific IgG across the placenta increased with time from vaccination (19) and mirrors previous data observed in infection (27, 46, 48). As more women vaccinated in the second trimester and earlier go on to deliver, it will be important to determine whether the COVID-19 vaccines, which induce immune responses to a pathogen previously never seen by their immune systems, may require administration even earlier in pregnancy than vaccines that evoke recall responses to offer optimal immunity to the neonate. In addition to antibodies transferred through the placenta, antibodies transferred through breastmilk have been shown to play a role in the protection against respiratory infections during early life (49–51). Interestingly, breastmilk antibodies are highly dependent on the second dose of the vaccine to boost the transfer of functional, FcR-binding antibodies. Previous work has shown that IgG antibodies in breastmilk may provide protection against viral infection in early life (52–54). Defining the mechanism driving breastmilk transfer of IgG antibodies could lay the foundation for designing next generation vaccines able to provide global protection for infants following birth.

Despite the delayed kinetics and functional antibody responses in pregnant versus non-pregnant women, pregnant and lactating women generated distinct immune responses when they were vaccinated with mRNA-1273 as compared to BNT162b2. These differential profiles were accompanied by a more restrictive, coordinated humoral immune response in women vaccinated with mRNA-1273 as compared to women who received BNT162b2. Whether this is related to differences in the dose, the lipid carriers, or the dosing window (4 versus 3 weeks) remains unclear. The extra week prior to boosting may provide the time needed for the humoral immune response to mature, resulting in more functional antibody profiles. Whether the functional advantage described here in mRNA-1273-generated humoral immune profile results in improved clinical protection against COVID-19 remains to be determined. Critically, optimal dosing and intervals may vary across populations and should be based on empirical data, strongly arguing for the importance of research in pregnancy and lactation to protect this vulnerable population who are often neglected during vaccine development. Given the differences observed in real-world efficacy across the mRNA-1273 and BNT162b2 vaccines, demonstrating 76% and 42% efficacy against the Delta variant of concern (55), the impact of the different Fc-profiles observed here may help explain this observation and provide critical insight on correlates of immunity for pregnant and non-pregnant populations.

There are several limitations to this study. First, since these sample were collected during the first months after the EUA for both vaccines, this study only included healthcare workers from a single city, preventing the ability to dissect differences in vaccine response across multiple demographics. Additionally, only women that were vaccinated in the third trimester gave birth in time to study cord blood, limiting our ability to understand opportunities to leverage transfer differences across COVID-19 vaccines across gestation. A further limitation is that some of the maternal blood samples were not collected at the time of delivery; thus observed differences between maternal and cord blood samples may be influenced in part by timing since vaccination. Moreover, although there was some variability in post-boost sampling across the individuals, the average time since boost to collection was similar across pregnant, lactating and non-pregnant women, offering an opportunity to compare profiles across the groups. This difference in the timing of collection of samples after booster vaccination could account for some observed differences between the three populations across the vaccines. A subset of the population were only collected at either the post-prime or post-boost timepoints, due to concerns in the frequency of hospital visits among some pregnant women. Thus, both an unmatched analysis was performed for the larger group and a matched analysis was performed in women that participated in both visits, both showing the same differences across Fc-receptor binding and Fc-functions, providing the first insights on the impact of pregnancy on shaping Fc-effector function, and the ability of distinct mRNA vaccines to shape antibody quality. Yet, future studies able to sample larger numbers of pregnant women vaccinated across the trimesters and following birth will offer an unparalleled opportunity to dissect the unique immunology of pregnancy and define additional strategies to develop vaccines aimed at specifically leveraging the unique biology of pregnancy and lactation with the goal to fully protect pregnant women against COVID-19 and other respiratory pathogens in the future.

Overall, our data have demonstrated that although antibody titers are similar, pregnant and lactating women respond to vaccination in qualitatively and kinetically distinct manners compared to non-pregnant women. Although this study largely profiled responses in women vaccinated later in pregnancy, these data point to the importance of profiling women receiving COVID-19 vaccines throughout pregnancy to begin to understand how distinct platforms, vaccines, populations, and timing affect the quality and quantity of immunity induced across the mother:fetal dyad. Collectively, these data highlight to the importance of defining the immunology of pregnancy to develop evidence-based recommendations for vaccine recommendations and to help inspire the development of vaccines and therapeutics that may act more effectively for this unique population where optimal immunological responses are necessary to protect both mother and fetus.

## Materials and Methods:

### Study Design

The goal of this study was to determine if pregnant and lactating women induce different antibody Fc profiles compared to non-pregnant women and to measure the transfer of vaccine-induced antibodies to neonates. Women at two tertiary care centers were approached for enrollment in an institutional review board (IRB)-approved (protocol #2020P003538) COVID-19 pregnancy and lactation biorepository study between December 17, 2020 and February 23, 2021. Eligible women were: (n=84 pregnant; (n=31) lactating; or (n= 16) non-pregnant and of reproductive age (18–45); greater than or equal to 18 years old, able to provide informed consent, and receiving the COVID-19 vaccine. Eligible study participants were identified by practitioners at the participating hospitals or were self-referred. A study questionnaire was administered to assess pregnancy and lactation status, history of prior SARS-CoV-2 infection, timing of COVID-19 vaccine doses, type of COVID-19 vaccine received (BNT162b2 Pfizer/BioNTech (n= 65) or mRNA-1273 Moderna/NIH (n= 66)).

Blood and breastmilk from lactating women were collected at the post-prime timepoint (at the time of second vaccine dose, 3 to 4 weeks post initial vaccination), the post-boost timepoint (2 to 5.5 weeks following the second vaccine dose), and at delivery (for pregnant participants who delivered during the study timeframe). Due to collection constraints, some individuals only contributed samples at the post-prime or post-boost timepoint, but not both. Umbilical cord blood was also collected at delivery for pregnant participants. The post-boost timepoint reflects full antibody complement, achieved one week after Pfizer/BioNTech BNT162b2 vaccination and two weeks after Moderna/NIH mRNA-1273 vaccination. Blood was collected by venipuncture (or from the umbilical vein following delivery for cord blood) into serum separator and EDTA tubes. Blood was centrifuged at 1000g for 10 minutes at room temperature. Serum and plasma were aliquoted into cryogenic vials and stored at −80°C.

For systems serology analysis, two replicates were performed for every experiment, and the data represents an average of the replicates. Experimenters were kept blinded during experimentation and only unblinded after all experiments were concluded. Samples for which individuals had previously tested positive for SARS-CoV-2 were excluded from analysis. For functional assays, samples were excluded due to sample volume constraints.

### Antigens

SARS-CoV-2 antigens used for functional and Luminex based assays included SARS-CoV-2 RBD (Sino Biological), SARS-CoV-2 spike protein (LakePharma) and SARS-CoV-2 N (Aalto Bio Reagents). Additional antigens included a mix of HA A/Michigan/45/2015 (H1N1), HA A/Singapore/ INFIMH-16–0019/2016 (H3N2), and HA B/Phuket/3073/2013 (Immunetech). Antigen was biotinylated using Sulfo-NHS-LC-LC biotin (Thermo Fisher Scientific) and desalted using Zeba Columns (Thermo Fisher Scientific).

### Primary Cells

Human neutrophils and NK cells were isolated from fresh peripheral blood. Peripheral blood was collected by the Massachusetts General Hospital (MGH) Blood Bank or by the Ragon Institute from healthy volunteers. All volunteers were over 18 years of age and gave signed consent. Samples were deidentified before use. The study was approved by the MGH Institutional Review Board. Human neutrophils were maintained in R10 media (RPMI-1640 (Sigma Aldrich) media supplemented with 10% fetal bovine serum (FBS) (Sigma Aldrich), 5% penicillin/streptomycin (Corning, 50 μg/mL), 5% L-glutamine (Corning, 4 mM), 5% HEPES buffer (pH 7.2) (Corning, 50 mM)) and grown at 37ºC, 5% CO2 for the duration of the assay. Human NK cells were rested overnight in R10 media supplemented with 2 ng/mL interleukin (IL)-15 at 37ºC, 5%CO2 and maintained in R10 media for the duration of the assay.

### Antibody-dependent cellular phagocytosis

THP-1 cells (American Type Culture Collection, ATCC) were used in phagocytic assays were grown in RPMI-1640 (Sigma Aldrich) media supplemented with 10% fetal bovine serum (FBS) (Sigma Aldrich), 5% penicillin/streptomycin (Corning, 50 μg/mL), 5% L-glutamine (Corning, 4mM), 5% HEPES buffer (pH 7.2) (Corning, 50 mM) and 0.5% 2-Mercaptoethanol (Gibco, 275 μM). Cells were maintained at a concentration of 2.5×10^5^ cells/ml. Antibody-dependent cellular phagocytosis was measured using a flow cytometry-based phagocytic assay (56). Briefly, 1.0 μm, yellow-green fluorescent (505/515) FluoSpheres NeutrAvidin (Thermo Fisher Scientific) were coated with biotinylated spike protein or HA, incubated with serum samples diluted 1:100, and breastmilk diluted at 1:10. The ability of samples to drive uptake of antigen-coated beads by THP-1 cells after overnight incubation was assessed by flow cytometry using the iQue (Intellicyt). Phagocytic scores were calculated as follows: (% yellow-green+ cells x yellow-green MFI)/100. A pool of serum samples collected from SARS-CoV-2 infected patients was used as a positive control, and a pool of serum samples from non-infected patients or 1x phosphate-buffered saline (PBS) alone was used as a negative control. Samples were run in duplicate. Data reported is an average of all data points collected.

### Antibody-dependent neutrophil phagocytosis

Antibody-dependent neutrophil phagocytosis was measured by a flow cytometry-based assay (57). Briefly, serum samples were diluted 1:100, and breastmilk was diluted at 1:10. Samples were then allowed to form immune complexes with 1.0 μm yellow-green fluorescent (505/515 nm) FluoSpheres NeutrAvidin (Thermo Fisher Scientific) beads coated with biotinylated antigen spike protein or HA antigens. White blood cells were isolated from whole blood using ACK Lysing Buffer (Thermo Fisher Scientific) to lyse red blood cells at room temperature (1:10). After lysis, remaining cells were counted and resuspended at 2.5×10^5^ cells/ml in RPMI-1640 (Sigma Aldrich) media supplemented with 10% fetal bovine serum (FBS) (Sigma Aldrich), 5% penicillin/streptomycin (Corning, 50 μg/mL), 5% L-glutamine (Corning, 4 mM), and 5% HEPES buffer (pH 7.2) (Corning, 50 mM). Cells were then incubated with bead and antibody mixture for 1 hour at 37ºC and then stained with Pacific Blue-conjugated anti-CD66b (BioLegend, clone: UCH71, 2 μg/mL) in PBS for 15 minutes at room temperature in the dark. Finally, cells were fixed in 4% paraformaldehyde (PFA) and phagocytosis of beads by CD66b^+^ cells was measured by flow cytometry using the iQue (Intellicyt). A pool of serum samples collected from SARS-CoV-2 infected patients was used as a positive control, and a pool of serum samples from non-infected patients or 1xPBS alone was used as a negative control. Samples were run in duplicate. Data reported is an average of data points collected from two donors.

### Antibody-dependent NK cell degranulation

Antibody-dependent NK cell degranulation was measured as described previously (58). Briefly, 96-well enzyme-linked immunosorbent assay (ELISA) plates were coated with 2 g/ml spike protein or HA protein and incubated at 37ºC for 2 hours and blocked with 5% bovine serum albumin (BSA) at 4ºC overnight. NK cells were isolated from whole blood from healthy donors (as mentioned above) by negative selection using RosetteSep (STEMCELL Technologies) then separated using a ficoll gradient. NK cells were rested overnight in RPMI-1640 (Sigma Aldrich) media supplemented with 10% fetal bovine serum (FBS) (Sigma Aldrich), 5% penicillin/streptomycin (Corning, 50 μg/mL), 5% L-glutamine (Corning, 4 mM), and 5% HEPES buffer (pH 7.2) (Corning, 50 mM) media supplemented with 2 ng/mL IL-15. Serum samples were diluted 1:50 and breastmilk at 1:5. After blocking, samples were incubated with antigen-coated plates for 2 hours at 37°C to allow immune complexes to form. After the 2 hour immune complex incubation, NK cells were mixed with anti-CD107a–phycoerythrin (PE)–Cy5 (BD Biosciences, lot # 0149826, 1:1000 dilution), brefeldin A (10 μg/ml) (Sigma-Aldrich), and GolgiStop (BD Biosciences), and added to antigen/antibody-coated plates for 5 hours at 37°C. The cells were stained for surface markers for 15 minutes at room temperature in PBS with anti-CD3 Pacific Blue (BD Biosciences, clone G10F5, 375 ng/mL)), anti-CD16 allophycocyanin (APC)-Cy5 (BD Biosciences, clone 3G8, 1:10000 dilution), and anti-CD56 PE-Cy7 (BD Biosciences, clone B159, 1:10000 dilution) and fixed with PermA (Life Technologies) for 15 minutes at room temperature. NK cells were then permeabilized with Perm B (Life Tech) and stained with anti-MIP-1β PE (BD Biosciences, 4 μg/mL) for 15 minutes at room temperature. The cells were then analyzed by flow cytometry on the iQue (Intellicyt). NK cells were gates as CD3^−^, CD16^+^, CD56^+^ cells and NK cell activity was determined as the percent of NK cells positive for CD107a or MIP-1β. Antibody-dependent NK cell activation (ADNKA) was only performed on post-boost samples and cord blood.

### Luminex

Serum samples were run in a customized Luminex assay to quantify the relative concentration of antigen-specific antibody isotype and subclass profiles. Carboxylated magplex-microspheres (Luminex) were coupled to antigens SARS-CoV-2 RBD (Sino Biological), SARS-CoV-2 S (Lake Pharma), SARS-CoV-2 N (Aalto Bio Reagents). SARS-CoV-2 S1 (Sino Biological), SARS-CoV-2 S2 (Sino Biological), pertussis pertactin (List Reagents) and a mix of HA A/Michigan/45/2015 (H1N1), HA A/Singapore/ INFIMH-16–0019/2016 (H3N2), B/Phuket/3073/2013 (Immunetech), using covalent NHS-ester linkages by EDC and NHS (Thermo Fisher Scientific) as described previously (59). To form immune complexes, appropriately diluted serum (1:100 for IgG2/3, 1:500 for IgG1, and 1:1000 for all other FcRs) and breastmilk (1:5 for all detectors), was added to the antigen-coupled microspheres, and plates were incubated overnight at 4ºC, shaking at 700 rpm. The following day, plates were washed with 0.1% BSA 0.02% Tween-20. PE-coupled mouse anti-human detection antibodies (Southern Biotech) were used to detect antigen-specific antibody binding. For the detection of FcR binding, Avi-Tagged FcRs (Duke Human Vaccine Institute) were biotinylated using BirA500 kit (Avidity) per manufacturer’s instructions. Biotinylated FcRs were tagged with PE and added to immune complexes. Fluorescence was acquired using an Intellicyt iQue, and relative antigen-specific antibody titer and FcR binding is reported as Median Fluorescence Intensity (MFI).

### Statistical analysis

For univariate data analysis, statistics were calculated using GraphPad Prism version 8.0. For functional assays, univariate analysis was performed comparing the three groups of women within each timepoint and between timepoints for each group. For antibody isotype titers and FcR-binding, data was log10-transformed prior to plotting and statistical analysis. Significance was determined by a one-way ANOVA with followed by posthoc Tukey’s multiple comparison test. P-values were then corrected for multiple comparisons using the Bejamini-Hochberg procedure. Breastmilk data was dilution corrected before transfer ratio analysis. For comparisons between mother and either cord or breastmilk, Wilcoxon-matched pairs signed rank test was performed. To determine transfer ratios the cord serum or breastmilk value was divided by the respective mother’s value for each assay.

Multivariate analyses were performed in R (version 4.0.0) and Python (version 3.9.1). The data was centered and scaled. For principal component analysis (PCA), an unsupervised dimensionality reduction technique, and classification models, data was log10-transformed prior to centering and scaling. For classification models, LASSO feature selection (60) was performed to identify significant features using the “select_lasso” function in systemseRology R package (v1.0) (https://github.com/LoosC/systemsseRology). LASSO aims to minimize sum of the residual sum of squares and the absolute value of the regression coefficients with a tuning parameter. The tuning parameter for LASSO was defined using 5-fold cross-validation. LASSO selection was performed 100 times, and only features that were chosen in 80% of the repetitions were used to build the downstream models. Partial least squares discriminant analysis (PLSDA) was performed using the LASSO-selected features. Multi-level partial least squares discriminant analysis (mPLSDA) (61) was performed for classification of maternal and cord samples.

Specifically, PLSDA builds a latent variable that linearly combines features that provide the maximum variance between the outcome, generating a model with the lowest mean squared error (MSE). mPLSDA corrects for the paired profile of the maternal and cord serology data by subtracting the effects of matched maternal and cord features to focus on the difference within matched dyads. Model performance was evaluated by five-fold cross-validation. To evaluate model robustness, control models with permuted classifier labels or with random features were built 50 times. These control models were then cross-validated 10 times to determine model accuracy. P values were calculated as the probability that the true value was within the control distributions. For correlation networks, antibody features that had Bonferroni-adjusted p-values < 0.05 and a spearman |r|>0.75 were visualized within the networks.

## Supplementary Material

FigureS1 Figure S1. Similar antibody titers are induced in pregnant and lactating women following boost. (**A and B**) The bar plots show the loadings of the least absolute shrinkage operator (LASSO)-selected features along principal component (PC) 2 for post-prime (A) and post-boost (B) for the principal component analyses (PCAs) shown in Fig. 1A and 1B, respectively. FcγR, Fcγ receptor; RBD, receptor binding domain.
(**C and D**). The violin plots show the FcγR-binding (A) and antibody-dependent cellular phagocytosis (ADCP), antibody-dependent neutrophil phagocytosis (ADNP) activity against spike (B) for non-pregnant, pregnant, and lactating women 3 to 4 weeks post-prime vaccination (1) and 2 to 5.5 weeks post-boost vaccination (2) (non-pregnant n = 14, pregnant n = 29, lactating n = 11). Only matched samples in which there was a matched post-prime and post-boost sample were included in the analysis. The filled dots show the titer for women who received the mRNA-1273 vaccine, and outlines show the titer for women who received the BNT162b2 vaccine. Data are presented as median +/− IQR. Significance was determined by a one-way ANOVA with followed by posthoc Tukey’s multiple comparison test. P-values were then corrected for multiple comparisons using the Bejamini-Hochberg procedure. * p <0.05, ** p < 0.01,*** p < 0.001, ns, not significant, MFI median fluorescence intensity.

FigureS2 Figure S2. Third-term vaccination results in an inefficient transfer of antibodies to the neonate, dependent on the time since vaccination to delivery. **(A)** The dot plots show the IgG1, IgG3, Fcγ2a-binding and FcγR3a-binding titer against hemagglutinin (HA) for maternal (M) and cord (C) blood. Lines connect maternal:cord dyads (n = 8). Significance was determined by Wilcoxon-matched pairs signed rank test. * p <0.05.
**(B)** The dot plots show the antibody-dependent cellular phagocytosis (ADCP), antibody-dependent neutrophil phagocytosis (ADNP) and antibody-dependent natural killer cell activation (ADNKA) (percent CD107a^+^ and MIP-1β+) functional titer against HA for maternal (M) and cord (C) blood (n = 8). Lines connect maternal:cord dyads. Significance was determined by Wilcoxon-matched pairs signed rank test. No significant differences were observed.
**(C)** The scatter plot shows the cord:maternal transfer ratios of spike protein-specific antibodies at the time of delivery for IgG1, IgG2, IgG3, FcγR2B, and FcγR3A versus the time post second dose.
**(D)** The scatter plot shows the cord:maternal transfer ratios of spike protein-specific antibodies at the time of delivery for ADNP, ADCP, CD107a and MIP-1β versus the time post second dose.

FigureS3 Figure S3. Matched analysis shows that a second vaccine dose results in an induction of functional antibodies in serum and transfer of functional antibodies to breastmilk. **(A and B)** A multi-level PCA was built on LASSO-selected SARS-CoV-2-specific features 3 to 4 weeks post-prime (purple) and 2–5.5 weeks post boost (blue) in lactating women (n=11) (A) and breastmilk (n=11) (B). Only samples for which there was a matched post-prime and post-boost sample pair were included in the analysis. The ellipses represent the 95% confidence interval for each group. The heatmaps show the contribution of each feature along each principal component (PC). The color of the heatmap indicates in which group each feature is enriched. A blue heatmap indicates that the features were only enriched in post-boost samples.

FigureS4 Figure S4. Booster vaccination results in robust transfer of antibodies in breastmilk. **(A)** The violin plots show the levels of ADCP, ADNP, and ADNKA (percent CD107a^+^ and MIP-1β+) activity in breastmilk at 3 to 4 weeks post-prime vaccination (n = 29, pink) and at 2 to 5.5 weeks post-boost vaccination (n = 15, blue). Data are presented as median±IQR. Significance was determined by a Mann-Whitney test. ** p < 0.01, *** p < 0.001. No asterisk indicates no significance was observed.
**(B)** The dot plots show the IgG1, IgG3, IgA, IgM, FcγR2a-binding, FcγR2b-binding and FcγR3a-binding titers against SARS-CoV-2 spike post-boost vaccination in maternal serum (M) and breastmilk (BM). Lines connect matched maternal serum:breastmilk dyads (n = 13). Significance was determined by Wilcoxon-matched pairs signed rank test. *** p < 0.001.
**(C)** The dot plots show the transfer ratio (breastmilk:serum) of ADNKA (CD107a and MIP-1b) activity at the post-boost timepoint. The dotted horizontal line indicates a transfer ratio of 1. The horizontal bars indicate the median of each group. Significance was determined by Wilcoxon-matched pairs signed rank test. No significant differences were observed.

FigureS5 Figure S5. mRNA-1273 and BNT162b2-induced antibody profiles are similar after prime vaccination but diverge after boosting. **(A)** A LASSO partial least squares-discriminant analysis (PLSDA) model was built on post-prime vaccination data from all groups. The dot plot (left) shows the scores for each sample, with each dot representing a sample. The ellipses represent the 95% confidence interval for each group. The bar plot (right) shows the loadings of each LASSO-selected feature, where the color marks the group enrichment.
**(B)** The spider plot shows scaled values of each given antibody feature in each vaccine group.
**(C)** A LASSO PLSDA model was built using V2 data from only non-pregnant, pregnant or lactating women. The dot plot shows the scores for each sample, with each dot representing a sample. The ellipses represent the 95% confidence interval for each group. The bar plot shows the loadings of each LASSO-selected feature, where the color marks the group enrichment.
**(D)** The violin plots show differences in the top LASSO-selected features from Fig. 4A in non-pregnant (n = 8 for BNT162b2, n = 6 for mRNA-1273), pregnant (n = 17 for BNT162b2, n = 19 for mRNA-1273), and lactating (n = 5 for BNT162b2, n = 8 for mRNA-1273) women given either the mRNA-1273 (M, filled dots) or BNT162b2 (P, outline dots). Data are presented as median±IQR. Significance was only calculated between groups in the same time point and was determined by a one-way ANOVA followed by posthoc Šidák’s multiple comparison test. P-values were then corrected for multiple comparisons using the Bejamini-Hochberg procedure. No statistically significant differences were observed.

Data File Zip

## Figures and Tables

**Table 1.** Cohort Demographic Information. SD, standard deviation; n, number of participants; IQR, interquartile range.

Characteristic	Nonpregnant	Pregnant	Lactating
Age, mean (SD)	38.4 (8.3)	34.1 (3.3)	34.6 (2.6)
Sample n, post-prime	13	64	28
Sample n, post-boost	14	36	13
Matched n (both post-prime and post-boost)	12	29	11
Vaccine type post-prime			
BNT162b2	6	32	15
mRNA-1273	7	32	13
Vaccine type post-boost			
BNT162b2	8	17	5
mRNA-1273	6	19	8
Days from dose 1 to post-prime collection, median (IQR)	28 (27–29)	28 (22–29.5)	28 (23–30)
Days from dose 1 to post-prime collection, median (IQR), BNT162b2	25.5 (23.25–27.75)	22 (21–24.75)	22.5 (21–26.5)
Days from dose 1 to post-prime collection, median (IQR), mRNA-1273	29 (28–29.25)	29 (28–31)	30 (28–34.5)
Days from dose 1 to post-boost collection, median (IQR)	49 (47.25–51.5)	49.5 (43.75–56.25)	49 (43–49.5)
Days from dose 1 to post-boost collection, median (IQR), BNT162b2	51 (49–55)	57 (50–58)	55 (50–59)
Days from dose 1 to post-boost collection, median (IQR), mRNA-1273	45.5 (44–47.75)	47.5 (43.25–49.75)	44 (43–49)
Days from dose 2 to post-boost collection, median (IQR)	26 (19–30.25)	20 (15–31.75)	21 (14–29)
Days from dose 2 to post-boost collection, median (IQR), BNT162b2	29.5 (27.5–33.5)	34 (14.75–37)	34 (29–34)
Days from dose 2 to post-boost collection, median (IQR), mRNA-1273	17.5 (14.5–19)	17 (15–21)	14.5 (14–18)
Gestational Age at post-prime, median (IQR)	-	23.2 (16.3–32.1)	-

## References

[1] Zambrano LD, Ellington S, Strid P, Galang RR, Oduyebo T, Tong VT, Woodworth KR, Nahabedian JF, Azziz-Baumgartner E, Gilboa SM, Meaney-Delman D, Akosa A, Bennett C, Burkel V, Chang D, Delaney A, Fox C, Griffin I, Hsia J, Krause K, Lewis E, Manning S, Mohamoud Y, Newton S, Neelam V, Olsen EO, Perez M, Reynolds M, Riser A, Rivera M, Roth NM, Sancken C, Shinde N, Smoots A, Snead M, Wallace B, Whitehill F, Whitehouse E, Zapata L, Update: Characteristics of Symptomatic Women of Reproductive Age with Laboratory-Confirmed SARS-CoV-2 Infection by Pregnancy Status — United States, January 22–October 3, 2020, MMWR. Morb. Mortal. Wkly. Rep. 69, 1641–1647 (2020). 33151921 10.15585/mmwr.mm6944e3 PMC7643892

[2] Lokken EM, Taylor GG, Huebner EM, Vanderhoeven J, Hendrickson S, Coler B, Sheng JS, Walker CL, McCartney SA, Kretzer NM, Resnick R, Kachikis A, Barnhart N, Schulte V, Bergam B, K K, Albright C, Larios V, Kelley L, Larios V, Emhoff S, Rah J, Retzlaff K, Thomas C, Paek BW, Hsu RJ, Erickson A, Chang A, Mitchell T, Hwang JK, Gourley R, Erickson S, Delaney S, Kline CR, Archabald K, Blain M, Lacourse SM, Adams Waldorf KM, Higher SARS-CoV-2 Infection Rate in Pregnant Patients., Am. J. Obstet. Gynecol. (2021), doi:10.1016/j.ajog.2021.02.011. PMC7884918 33607103

[3] Riley LE, Jamieson DJ, Inclusion of Pregnant and Lactating Persons in COVID-19 Vaccination Efforts, Ann. Intern. Med., 9–11 (2021). 10.7326/M21-0173 PMC7875205 33493013

[4] Bianchi DW, Kaeser L, Cernich AN, Involving Pregnant Individuals in Clinical Research on COVID-19 Vaccines, Jama 20892, 2–3 (2021). 10.1001/jama.2021.1865 PMC10080263 33566088

[5] Beigi RH, Krubiner C, Jamieson DJ, Lyerly AD, Hughes B, Riley L, Faden R, Karron R, The need for inclusion of pregnant women in COVID-19 vaccine trials, Vaccine 39, 868–870 (2021). 33446385 10.1016/j.vaccine.2020.12.074 PMC7798437

[6] Abu-Raya B, Michalski C, Sadarangani M, Lavoie PM, Maternal Immunological Adaptation During Normal Pregnancy, Front. Immunol. 11, 1–18 (2020). 33133091 10.3389/fimmu.2020.575197 PMC7579415

[7] Razzaghi H, Kahn KE, Black CL, Lindley MC, Jatlaoui TC, Fiebelkorn AP, Havers FP, D’Angelo DV, Cheung A, Ruther NA, Williams WW, Influenza and Tdap Vaccination Coverage Among Pregnant Women — United States, April 2020, MMWR. Morb. Mortal. Wkly. Rep. 69, 1391–1397 (2020). 33001873 10.15585/mmwr.mm6939a2 PMC7537555

[8] Adhikari EH, Spong CY, COVID-19 Vaccination in Pregnant and LactatingWomen, JAMA 325, 1041 (2021). 33555297 10.1001/jama.2021.1658

[9] Kay AW, Blish CA, Immunogenicity and clinical efficacy of influenza vaccination in pregnancy, Front. Immunol. 6, 1–9 (2015). 26089824 10.3389/fimmu.2015.00289 PMC4455389

[10] Grohskopf LA, Alyanak E, Broder KR, Blanton LH, Fry AM, Jernigan DB, Atmar RL, Prevention and control of seasonal influenza with vaccines: Recommendations of the advisory committee on immunization practices-United States, 2020–21 influenza season, MMWR Recomm. Reports 69 (2020), doi:10.15585/MMWR.RR6908A1. PMC7439976 32820746

[11] Liang JL, Tiwari T, Moro P, Messonnier NE, Reingold A, Sawyer M, Clark TA, Prevention of pertussis, tetanus, and diphtheria with vaccines in the United States: Recommendations of the Advisory Committee on Immunization Practices (ACIP), MMWR Recomm. Reports 67 (2018), doi:10.15585/mmwr.rr6702a1. PMC5919600 29702631

[12] Havers FP, Moro PL, Hunter P, Hariri S, Bernstein H, Use of Tetanus Toxoid, Reduced Diphtheria Toxoid, and Acellular Pertussis Vaccines: Updated Recommendations of the Advisory Committee on Immunization Practices — United States, 2019, MMWR. Morb. Mortal. Wkly. Rep. 69, 77–83 (2020). 31971933 10.15585/mmwr.mm6903a5 PMC7367039

[13] Eberhardt CS, Blanchard-Rohner G, Lemaître B, Boukrid M, Combescure C, Othenin-Girard V, Chilin A, Petre J, De Tejada BM, Siegrist CA, Maternal immunization earlier in pregnancy maximizes antibody transfer and expected infant seropositivity against pertussis, Clin. Infect. Dis. 62, 829–836 (2016). 26797213 10.1093/cid/ciw027 PMC4787611

[14] Polack FP, Thomas SJ, Kitchin N, Absalon J, Gurtman A, Lockhart S, Perez JL, Pérez Marc G, Moreira ED, Zerbini C, Bailey R, Swanson KA, Roychoudhury S, Koury K, Li P, Kalina WV, Cooper D, Frenck RW, Hammitt LL, Türeci Ö, Nell H, Schaefer A, Ünal S, Tresnan DB, Mather S, Dormitzer PR, Şahin U, Jansen KU, Gruber WC, Safety and Efficacy of the BNT162b2 mRNA Covid-19 Vaccine, N. Engl. J. Med. 383 (2020), doi:10.1056/nejmoa2034577. PMC7745181 33301246

[15] Baden LR, El Sahly HM, Essink B, Kotloff K, Frey S, Novak R, Diemert D, Spector SA, Rouphael N, Creech CB, McGettigan J, Khetan S, Segall N, Solis J, Brosz A, Fierro C, Schwartz H, Neuzil K, Corey L, Gilbert P, Janes H, Follmann D, Marovich M, Mascola J, Polakowski L, Ledgerwood J, Graham BS, Bennett H, Pajon R, Knightly C, Leav B, Deng W, Zhou H, Han S, Ivarsson M, Miller J, Zaks T, Efficacy and Safety of the mRNA-1273 SARS-CoV-2 Vaccine, N. Engl. J. Med. 384 (2021), doi:10.1056/nejmoa2035389. PMC7787219 33378609

[16] Anderson EJ, Rouphael NG, Widge AT, Jackson LA, Roberts PC, Makhene M, Chappell JD, Denison MR, Stevens LJ, Pruijssers AJ, McDermott AB, Flach B, Lin BC, Doria-Rose NA, O’Dell S, Schmidt SD, Corbett KS, Swanson PA, Padilla M, Neuzil KM, Bennett H, Leav B, Makowski M, Albert J, Cross K, Edara VV, Floyd K, Suthar MS, Martinez DR, Baric R, Buchanan W, Luke CJ, Phadke VK, Rostad CA, Ledgerwood JE, Graham BS, Beigel JH, Safety and Immunogenicity of SARS-CoV-2 mRNA-1273 Vaccine in Older Adults, N. Engl. J. Med. 383 (2020), doi:10.1056/nejmoa2028436. PMC7556339 32991794

[17] Walsh EE, Frenck RW, Falsey AR, Kitchin N, Absalon J, Gurtman A, Lockhart S, Neuzil K, Mulligan MJ, Bailey R, Swanson KA, Li P, Koury K, Kalina W, Cooper D, Fontes-Garfias C, Shi P-Y, Türeci Ö, Tompkins KR, Lyke KE, Raabe V, Dormitzer PR, Jansen KU, Şahin U, Gruber WC, Safety and Immunogenicity of Two RNA-Based Covid-19 Vaccine Candidates, N. Engl. J. Med. 383 (2020), doi:10.1056/nejmoa2027906. PMC7583697 33053279

[18] Gray KJ, Bordt EA, Atyeo C, Alter G, Edlow AG, COVID-19 vaccine response inpregnant and lactating women: a cohort study, Am J Obs. Gynecol. (2021), doi:10.1016/j.ajog.2021.03.023. PMC7997025 33775692

[19] MITHAL LB, OTERO S, SHANES ED, GOLDSTEIN JA, MILLER ES, Cord Blood Antibodies following Maternal COVID-19 Vaccination During Pregnancy, Am. J. Obstet. Gynecol. (2021), doi:10.1016/j.ajog.2021.03.035. PMC8012273 33812808

[20] Schäfer A, Muecksch F, Lorenzi JCC, Leist SR, Cipolla M, Bournazos S, Schmidt F, Gazumyan A, Baric RS, Robbiani DF, Hatziioannou T, Ravetch JV, Bieniasz PD, Nussenzweig MC, Sheahan TP, Antibody potency, effector function and combinations in protection from SARS-CoV-2 infection in vivo, J. Exp. Med. 218 (2020), doi:10.1101/2020.09.15.298067. PMC7673958 33211088

[21] Zohar T, Loos C, Fischinger S, Atyeo C, Wang C, Slein MD, Burke J, Yu J, Feldman J, Hauser BM, Caradonna T, Schmidt AG, Cai Y, Streeck H, Ryan ET, Barouch DH, Charles RC, Lauffenburger DA, Alter G, Compromised Humoral Functional Evolution Tracks with SARS-CoV-2 Mortality, Cell 183, 1508–1519.e12 (2020). 33207184 10.1016/j.cell.2020.10.052 PMC7608014

[22] Zohar T, Loos C, Fischinger S, Atyeo C, Wang C, Slein MD, Burke J, Yu J, Feldman J, Hauser BM, Caradonna T, Schmidt AG, Cai Y, Streeck H, Ryan ET, Barouch DH, Charles RC, Lauffenburger DA, Alter G, Compromised Humoral Functional Evolution Tracks with SARS-CoV-2 Mortality, Cell 183, 1508–1519.e12 (2020). 33207184 10.1016/j.cell.2020.10.052 PMC7608014

[23] Yu J, Tostanosk LH, Peter L, Mercad NB, McMahan K, Mahrokhia SH, Nkolol JP, Liu J, Li Z, Chandrashekar A, Martine DR, Loos C, Atyeo C, Fischinger S, Burk JS, Slei MD, Chen Y, Zuiani A, Lelis FJN, Travers M, Habibi S, Pessaint L, Van Ry A, Blade K, Brown R, Cook A, Finneyfrock B, Dodson A, Teow E, Velasco J, Zahn R, Wegmann F, Bondzi EA, Dagotto G, Gebr MS, He X, Jacob-Dolan C, Kirilova M, Kordana N, Lin Z, Maxfiel LF, Nampanya F, Nityanandam R, Ventur JD, Wan H, Cai Y, Chen B, Schmid AG, Weseman DR, Bari RS, Alter G, Andersen H, Lewi MG, Barou DH, DNA vaccine protection against SARS-CoV-2 in rhesus macaques, Science (80-. ). 369 (2020), doi:10.1126/science.abc6284. PMC7243363 32434945

[24] Clements T, Rice TF, Vamvakas G, Barnett S, Barnes M, Donaldson B, Jones CE, Kampmann B, Holder B, Update on Transplacental Transfer of IgG Subclasses: Impact of Maternal and Fetal Factors, Front. Immunol. 11, 1–17 (2020). 33013843 10.3389/fimmu.2020.01920 PMC7516031

[25] Jennewein MF, Goldfarb I, Dolatshahi S, Cosgrove C, Noelette FJ, Krykbaeva M, Das J, Sarkar A, Gorman MJ, Fischinger S, Boudreau CM, Brown J, Cooperrider JH, Aneja J, Suscovich TJ, Graham BS, Lauer GM, Goetghebuer T, Marchant A, Lauffenburger D, Kim AY, Riley LE, Alter G, Fc Glycan-Mediated Regulation of Placental Antibody Transfer, Cell 178, 202–215.e14 (2019). 31204102 10.1016/j.cell.2019.05.044 PMC6741440

[26] Wilcox CR, Holder B, Jones CE, Factors affecting the FcRn-mediated transplacental transfer of antibodies and implications for vaccination in pregnancy, Front. Immunol. 8 (2017), doi:10.3389/fimmu.2017.01294. PMC5671757 29163461

[27] Atyeo C, Pullen KM, Bordt EA, Fischinger S, Burke J, Michell A, Slein MD, Loos C, Shook LL, Boatin AA, Yockey LJ, Pepin D, Meinsohn M-C, Phuong Nguyen NM, Chauvin M, Roberts D, Goldfarb IT, Matute JD, James KE, Yonker LM, Bebell LM, Kaimal AJ, Gray KJ, Lauffenburger D, Edlow AG, Alter G, Compromised SARS-CoV-2-specific placental antibody transfer, Cell (2020), doi:10.1016/j.cell.2020.12.027. PMC7755577 33476549

[28] Langel SN, Otero CE, Martinez DR, Permar SR, Maternal gatekeepers: How maternal antibody Fc characteristics influence passive transfer and infant protection, PLoS Pathog. 16, 1–8 (2020). 10.1371/journal.ppat.1008303 PMC7098569 32214394

[29] Gray KJ, Bordt EA, Atyeo C, Deriso E, Akinwunmi B, Young N, Medina Baez A, Shook LL, Cvrk D, James K, De Guzman R, Brigida S, Diouf K, Goldfarb I, Bebell LM, Yonker LM, Fasano A, Rabi SA, Elovitz MA, Alter G, Edlow AG, COVID-19 vaccine response in pregnant and lactating women: a cohort study, Am. J. Obstet. Gynecol. (2021), doi:10.1016/j.ajog.2021.03.023. PMC7997025 33775692

[30] Collier AY, McMahan K, Yu J, Tostanoski LH, Aguayo R, Ansel J, Chandrashekar A, Patel S, Apraku Bondzie E, Sellers D, Barrett J, Sanborn O, Wan H, Chang A, Anioke T, Nkolola J, Bradshaw C, Jacob-Dolan C, Feldman J, Gebre M, Borducchi EN, Liu J, Schmidt AG, Suscovich T, Linde C, Alter G, Hacker MR, Barouch DH, Immunogenicity of COVID-19 mRNA Vaccines in Pregnant and Lactating Women, JAMA (2021), doi:10.1001/jama.2021.7563. PMC8120446 33983379

[31] FDA, Vaccines and Related Biological Products Advisory Committee Meeting, 1–30 (2020).

[32] Vrbpac V, Document B, PFIZER-BIONTECH COVID-19 VACCINE (BNT162, PF-07302048) VACCINES AND RELATED BIOLOGICAL PRODUCTS ADVISORY COMMITTEE BRIEFING DOCUMENT MEETING DATE: 10 December 2020, 23 (2020).

[33] Jackson LA, Anderson EJ, Rouphael NG, Roberts PC, Makhene M, Coler RN, McCullough MP, Chappell JD, Denison MR, Stevens LJ, Pruijssers AJ, McDermott A, Flach B, Doria-Rose NA, Corbett KS, Morabito KM, O’Dell S, Schmidt SD, Swanson PA, Padilla M, Mascola JR, Neuzil KM, Bennett H, Sun W, Peters E, Makowski M, Albert J, Cross K, Buchanan W, Pikaart-Tautges R, Ledgerwood JE, Graham BS, Beigel JH, An mRNA Vaccine against SARS-CoV-2 — Preliminary Report, N. Engl. J. Med. 383, 1920–1931 (2020). 32663912 10.1056/NEJMoa2022483 PMC7377258

[34] Wilson E, Butcher EC, CCL28 controls immunoglobulin (Ig)A plasma cell accumulation in the lactating mammary gland and IgA antibody transfer to the neonate, J. Exp. Med. (2004), doi:10.1084/jem.20041069. PMC2211970 15381732

[35] Atyeo C, Alter G, The multifaceted roles of breast milk antibodies, Cell 184 (2021), doi:10.1016/j.cell.2021.02.031. 33740451

[36] Pazos M, Sperling RS, Moran TM, Kraus TA, The influence of pregnancy on systemic immunity, Immunol. Res. 54 (2012), doi:10.1007/s12026-012-8303-9. PMC7091327 22447351

[37] Robinson DP, Klein SL, Pregnancy and pregnancy-associated hormones alter immune responses and disease pathogenesisHorm. Behav. 62 (2012), doi:10.1016/j.yhbeh.2012.02.023. PMC3376705 22406114

[38] Ellington S, Strid P, Tong VT, Woodworth K, Galang RR, Zambrano LD, Nahabedian J, Anderson K, Gilboa SM, Characteristics of Women of Reproductive Age with Laboratory-Confirmed SARS-CoV-2 Infection by Pregnancy Status — United States, January 22–June 7, 2020, MMWR. Morb. Mortal. Wkly. Rep. (2020), doi:10.15585/mmwr.mm6925a1. PMC7316319 32584795

[39] Somerville LK, Basile K, Dwyer DE, Kok J, The impact of influenza virus infection in pregnancyFuture Microbiol. 13 (2018), doi:10.2217/fmb-2017-0096. 29320882

[40] Abu-Raya B, Michalski C, Sadarangani M, Lavoie PM, Maternal Immunological Adaptation During Normal Pregnancy Front. Immunol. 11 (2020), doi:10.3389/fimmu.2020.575197. PMC7579415 33133091

[41] Aghaeepour N, Ganio EA, Mcilwain D, Tsai AS, Tingle M, Van Gassen S, Gaudilliere DK, Baca Q, McNeil L, Okada R, Ghaemi MS, Furman D, Wong RJ, Winn VD, Druzin ML, El-Sayed YY, Quaintance C, Gibbs R, Darmstadt GL, Shaw GM, Stevenson DK, Tibshirani R, Nolan GP, Lewis DB, Angst MS, Gaudilliere B, An immune clock of human pregnancy, Sci. Immunol. 2 (2017), doi:10.1126/sciimmunol.aan2946. PMC5701281 28864494

[42] Dong Y, Dong Y, Mo X, Hu Y, Qi X, Jiang F, Jiang Z, Jiang Z, Tong S, Tong S, Tong S, Epidemiology of COVID-19 among children in China Pediatrics (2020), doi:10.1542/peds.2020-0702. 32179660

[43] Garg S, Kim L, Whitaker M, O’Halloran A, Cummings C, Holstein R, Prill M, Chai SJ, Kirley PD, Alden NB, Kawasaki B, Yousey-Hindes K, Niccolai L, Anderson EJ, Openo KP, Weigel A, Monroe ML, Ryan P, Henderson J, Kim S, Como-Sabetti K, Lynfield R, Sosin D, Torres S, Muse A, Bennett NM, Billing L, Sutton M, West N, Schaffner W, Talbot HK, Aquino C, George A, Budd A, Brammer L, Langley G, Hall AJ, Fry A, Hospitalization Rates and Characteristics of Patients Hospitalized with Laboratory-Confirmed Coronavirus Disease 2019 — COVID-NET, 14 States, March 1–30, 2020, MMWR. Morb. Mortal. Wkly. Rep. (2020), doi:10.15585/mmwr.mm6915e3. PMC7755063 32298251

[44] Liguoro I, Pilotto C, Bonanni M, Ferrari ME, Pusiol A, Nocerino A, Vidal E, Cogo P, SARS-COV-2 infection in children and newborns: a systematic review Eur. J. Pediatr. (2020), doi:10.1007/s00431-020-03684-7. PMC7234446 32424745

[45] Atyeo C, Pullen KM, Bordt EA, Fischinger S, Burke J, Michell A, Slein MD, Loos C, Shook LL, Boatin AA, Yockey LJ, Pepin D, Meinsohn MC, Nguyen NMP, Chauvin M, Roberts D, Goldfarb IT, Matute JD, James KE, Yonker LM, Bebell LM, Kaimal AJ, Gray KJ, Lauffenburger D, Edlow AG, Alter G, Compromised SARS-CoV-2-specific placental antibody transfer, Cell 184, 628–642.e10 (2021). 33476549 10.1016/j.cell.2020.12.027 PMC7755577

[46] Flannery DD, Gouma S, Dhudasia MB, Mukhopadhyay S, Pfeifer MR, Woodford EC, Triebwasser JE, Gerber JS, Morris JS, Weirick ME, McAllister CM, Bolton MJ, Arevalo CP, Anderson EM, Goodwin EC, Hensley SE, Puopolo KM, Assessment of Maternal and Neonatal Cord Blood SARS-CoV-2 Antibodies and Placental Transfer Ratios, JAMA Pediatr. (2021), doi:10.1001/jamapediatrics.2021.0038. PMC7846944 33512440

[47] Bordt EA, Shook LL, Atyeo C, Pullen KM, De Guzman RM, Meinsohn M-C, Chauvin M, Fischinger S, Yockey LJ, James K, Lima R, Yonker LM, Fasano A, Brigida S, Bebell LM, Roberts DJ, Pepin D, Huh JR, Bilbo SD, Li JZ, Kaimal A, Gray KJ, Lauffenburger D, Alter G, Edlow AG, Sexually dimorphic placental responses to maternal SARS-CoV-2 infection, Sci. Transl. Med. (2021), doi:10.1126/scitranslmed.abi7428. PMC8784281 34664987

[48] Edlow AG, Li JZ, Collier ARY, Atyeo C, James KE, Boatin AA, Gray KJ, A Bordt E, Shook LL, Yonker LM, Fasano A, Diouf K, Croul N, Devane S, Yockey LJ, Lima R, Shui J, Matute JD, Lerou PH, Akinwunmi BO, Schmidt A, Feldman J, Hauser BM, Caradonna TM, De la Flor D, D’Avino P, Regan J, Corry H, Coxen K, Fajnzylber J, Pepin D, Seaman MS, Barouch DH, Walker BD, Yu XG, Kaimal AJ, Roberts DJ, Alter G, Assessment of Maternal and Neonatal SARS-CoV-2 Viral Load, Transplacental Antibody Transfer, and Placental Pathology in Pregnancies During the COVID-19 Pandemic, JAMA Netw. open 3 (2020), doi:10.1001/jamanetworkopen.2020.30455. PMC7756241 33351086

[49] Schlaudecker EP, Steinhoff MC, Omer SB, McNeal MM, Roy E, Arifeen SE, Dodd CN, Raqib R, Breiman RF, Zaman K, IgA and Neutralizing Antibodies to Influenza A Virus in Human Milk: A Randomized Trial of Antenatal Influenza Immunization, PLoS One (2013), doi:10.1371/journal.pone.0070867. PMC3743877 23967126

[50] Henkle E, Steinhoff MC, Omer SB, Roy E, Arifeen SE, Raqib R, Breiman RE, Caulfield LE, Moss WJ, Zaman K, The effect of exclusive breast-feeding on respiratory illness in young infants in a maternal immunization trial in Bangladesh, Pediatr. Infect. Dis. J. (2013), doi:10.1097/INF.0b013e318281e34f. 23249922

[51] Nunes MC, Cutland CL, Jones S, Downs S, Weinberg A, Ortiz JR, Neuzil KM, Simões EAF, Klugman KP, Madhi SA, Efficacy of Maternal Influenza Vaccination Against All-Cause Lower Respiratory Tract Infection Hospitalizations in Young Infants: Results from a Randomized Controlled Trial, Clin. Infect. Dis. (2017), doi:10.1093/cid/cix497. PMC5848298 28575286

[52] Renegar KB, Small PA, Boykins LG, Wright PF, Role of IgA versus IgG in the Control of Influenza Viral Infection in the Murine Respiratory Tract, J. Immunol. (2004), doi:10.4049/jimmunol.173.3.1978. 15265932

[53] Mazur NI, Horsley NM, Englund JA, Nederend M, Magaret A, Kumar A, Jacobino SR, De Haan CAM, Khatry SK, Leclerq SC, Steinhoff MC, Tielsch JM, Katz J, Graham BS, Bont LJ, Leusen JHW, Chu HY, Breast milk prefusion F immunoglobulin g as a correlate of protection against respiratory syncytial virus acute respiratory illness, J. Infect. Dis. (2019), doi:10.1093/infdis/jiy477. PMC6284547 30107412

[54] Mabuka J, Nduati R, Odem-Davis K, Peterson D, Overbaugh J, HIV-specific antibodies capable of ADCC are common in breastmilk and are associated with reduced risk of transmission in women with high viral loads, PLoS Pathog. (2012), doi:10.1371/journal.ppat.1002739. PMC3375288 22719248

[55] Puranik A, Lenehan PJ, Silvert E, Niesen MJM, Corchado-Garcia J, O’Horo JC, Virk A, Swift MD, Halamka J, Badley AD, Venkatakrishnan AJ, Soundararajan V, Comparison of two highly-effective mRNA vaccines for COVID-19 during periods of Alpha and Delta variant prevalence, medRxiv, 2021.08.06.21261707 (2021).

[56] Ackerman ME, Moldt B, Wyatt RT, Dugast AS, McAndrew E, Tsoukas S, Jost S, Berger CT, Sciaranghella G, Liu Q, Irvine DJ, Burton DR, Alter G, A robust, high-throughput assay to determine the phagocytic activity of clinical antibody samples, J. Immunol. Methods (2011), doi:10.1016/j.jim.2010.12.016. PMC3050993 21192942

[57] Karsten CB, Mehta N, Shin SA, Diefenbach TJ, Slein MD, Karpinski W, Irvine EB, Broge T, Suscovich TJ, Alter G, A versatile high-throughput assay to characterize antibody-mediated neutrophil phagocytosis, J. Immunol. Methods (2019), doi:10.1016/j.jim.2019.05.006. PMC6620195 31132351

[58] Boudreau CM, Yu WH, Suscovich TJ, Talbot HK, Edwards KM, Alter G, Selective induction of antibody effector functional responses using MF59-adjuvanted vaccination, J. Clin. Invest. (2020), doi:10.1172/JCI129520. PMC6994146 31845904

[59] Brown EP, Dowell KG, Boesch AW, Normandin E, Mahan AE, Chu T, Barouch DH, Bailey-Kellogg C, Alter G, Ackerman ME, Multiplexed Fc array for evaluation of antigen-specific antibody effector profiles, J. Immunol. Methods (2017), doi:10.1016/j.jim.2017.01.010. PMC5333794 28163018

[60] Tibshirani R, Regression shrinkage and selection via the lasso: a retrospective, J. R. Stat.Soc. Ser. B (Statistical Methodol. 73 (2011), doi:10.1111/j.1467-9868.2011.00771.x.

[61] Westerhuis JA, van Velzen EJJ, Hoefsloot HCJ, Smilde AK, Multivariate paired data analysis: Multilevel PLSDA versus OPLSDA, Metabolomics (2010), doi:10.1007/s11306-009-0185-z. PMC2834771 20339442

