# Supplementary figures and images for: COVID-19 mRNA vaccines drive differential Fc-functional profiles in pregnant, lactating, and non-pregnant women
Source: Sci Transl Med. Author manuscript; Available in PMC 2022 May 4. (PMC9067624; doi:10.1126/scitranslmed.abi8631)

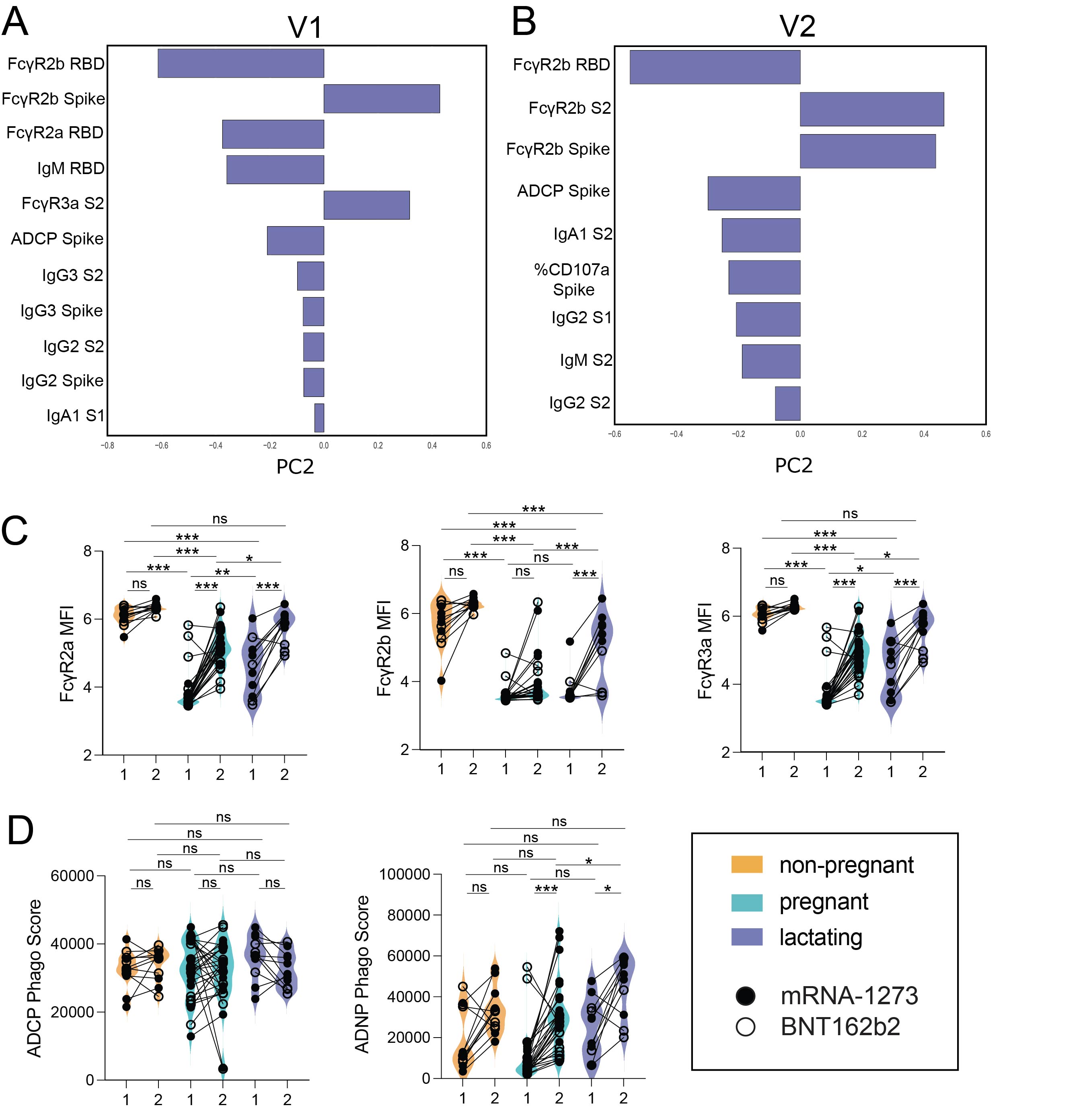

Supplement: FigureS1 — (A and B) The bar plots show the loadings of the least absolute shrinkage operator (LASSO)-selected features along principal component (PC) 2 for post-prime (A) and post-boost (B) for the principal component analyses (PCAs) shown in Fig. 1A and 1B, respectively. FcγR, Fcγ receptor; RBD, receptor binding domain. (C and D). The violin plots show the FcγR-binding (A) and antibody-dependent cellular phagocytosis (ADCP), antibody-dependent neutrophil phagocytosis (ADNP) activity against spike (B) for non-pregnant, pregnant, and lactating women 3 to 4 weeks post-prime vaccination (1) and 2 to 5.5 weeks post-boost vaccination (2) (non-pregnant n = 14, pregnant n = 29, lactating n = 11). Only matched samples in which there was a matched post-prime and post-boost sample were included in the analysis. The filled dots show the titer for women who received the mRNA-1273 vaccine, and outlines show the titer for women who received the BNT162b2 vaccine. Data are presented as median +/− IQR. Significance was determined by a one-way ANOVA with followed by posthoc Tukey’s multiple comparison test. P-values were then corrected for multiple comparisons using the Bejamini-Hochberg procedure. * p <0.05, ** p < 0.01,*** p < 0.001, ns, not significant, MFI median fluorescence intensity. [file NIHMS1758174-supplement-FigureS1.jpg]

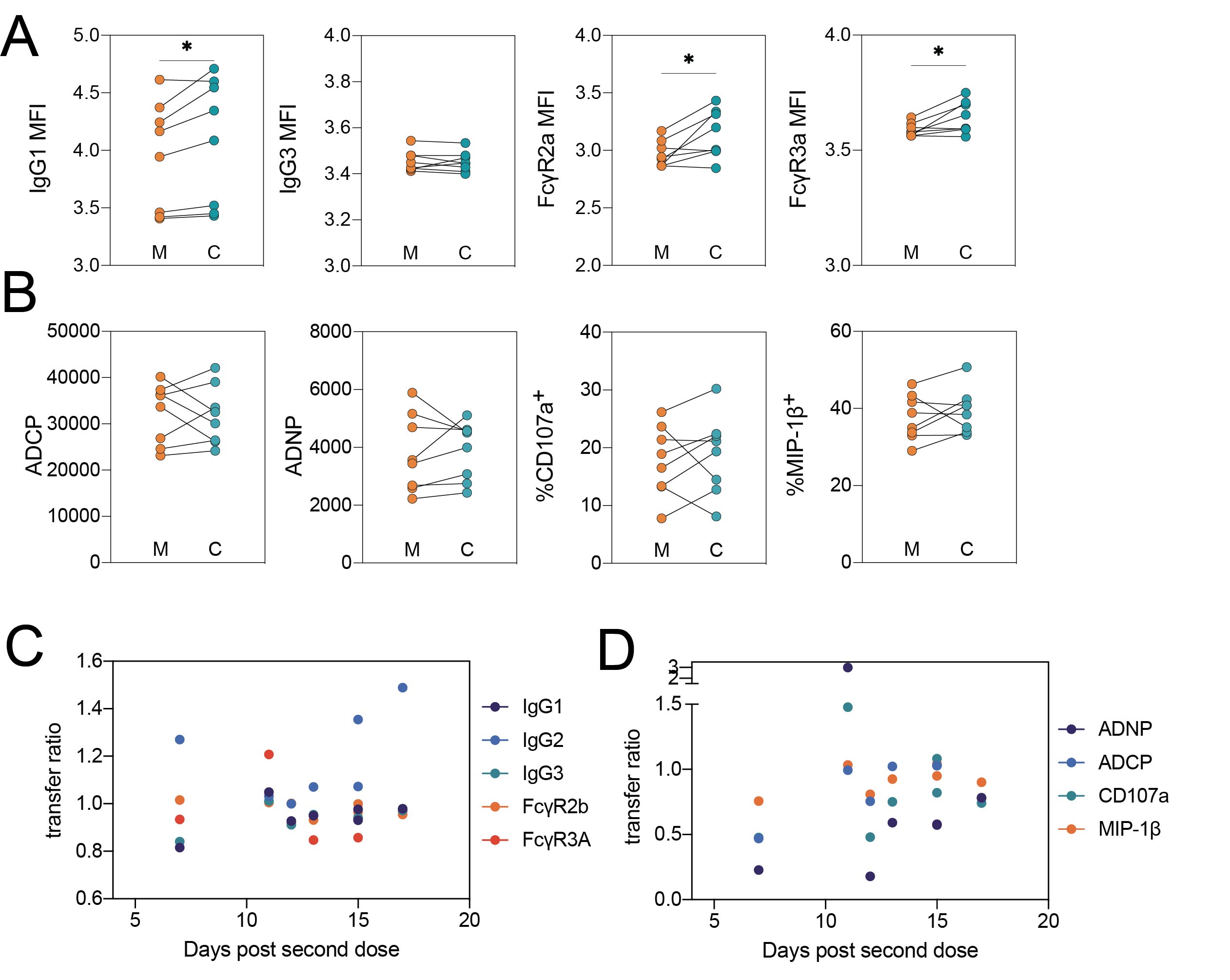

Supplement: FigureS2 — (A) The dot plots show the IgG1, IgG3, Fcγ2a-binding and FcγR3a-binding titer against hemagglutinin (HA) for maternal (M) and cord (C) blood. Lines connect maternal:cord dyads (n = 8). Significance was determined by Wilcoxon-matched pairs signed rank test. * p <0.05. (B) The dot plots show the antibody-dependent cellular phagocytosis (ADCP), antibody-dependent neutrophil phagocytosis (ADNP) and antibody-dependent natural killer cell activation (ADNKA) (percent CD107a+ and MIP-1β+) functional titer against HA for maternal (M) and cord (C) blood (n = 8). Lines connect maternal:cord dyads. Significance was determined by Wilcoxon-matched pairs signed rank test. No significant differences were observed. (C) The scatter plot shows the cord:maternal transfer ratios of spike protein-specific antibodies at the time of delivery for IgG1, IgG2, IgG3, FcγR2B, and FcγR3A versus the time post second dose. (D) The scatter plot shows the cord:maternal transfer ratios of spike protein-specific antibodies at the time of delivery for ADNP, ADCP, CD107a and MIP-1β versus the time post second dose. [file NIHMS1758174-supplement-FigureS2.jpg]

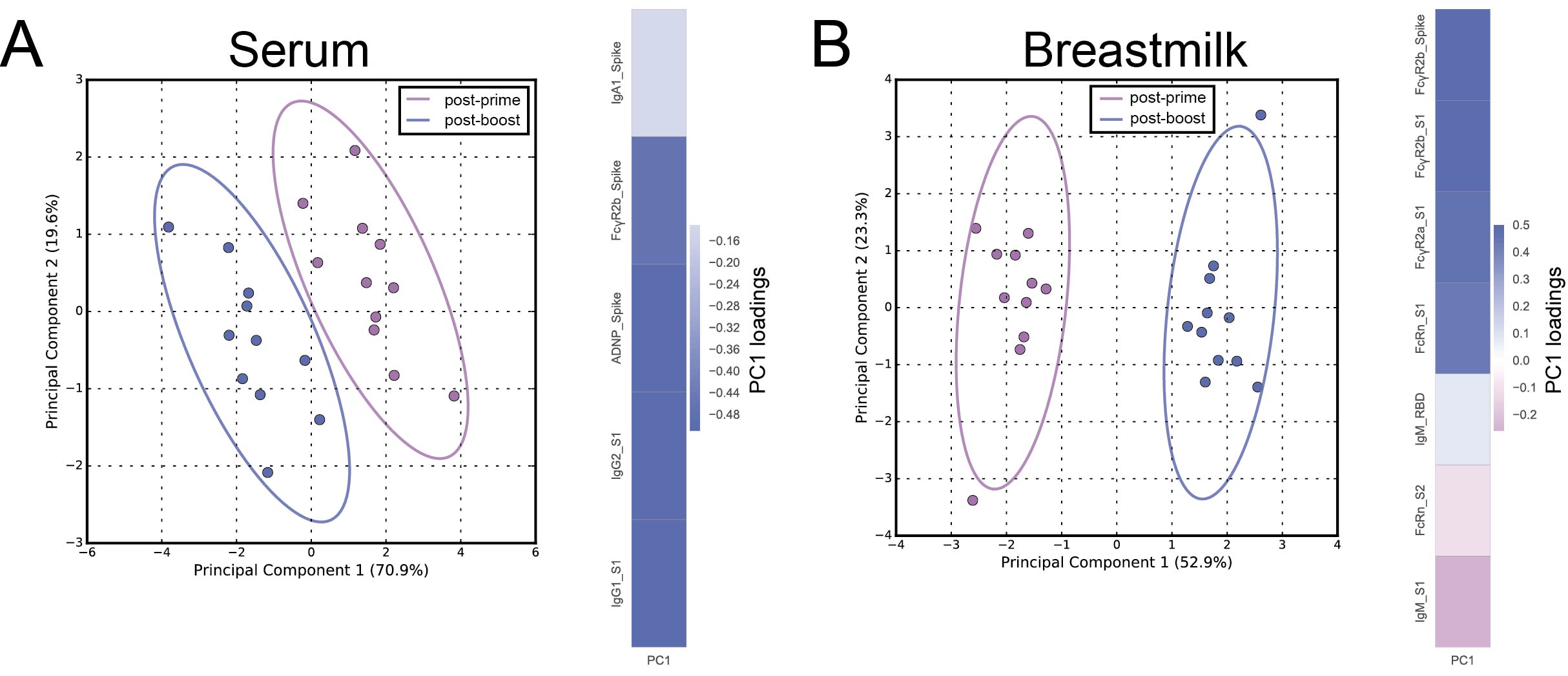

Supplement: FigureS3 — (A and B) A multi-level PCA was built on LASSO-selected SARS-CoV-2-specific features 3 to 4 weeks post-prime (purple) and 2–5.5 weeks post boost (blue) in lactating women (n=11) (A) and breastmilk (n=11) (B). Only samples for which there was a matched post-prime and post-boost sample pair were included in the analysis. The ellipses represent the 95% confidence interval for each group. The heatmaps show the contribution of each feature along each principal component (PC). The color of the heatmap indicates in which group each feature is enriched. A blue heatmap indicates that the features were only enriched in post-boost samples. [file NIHMS1758174-supplement-FigureS3.jpg]

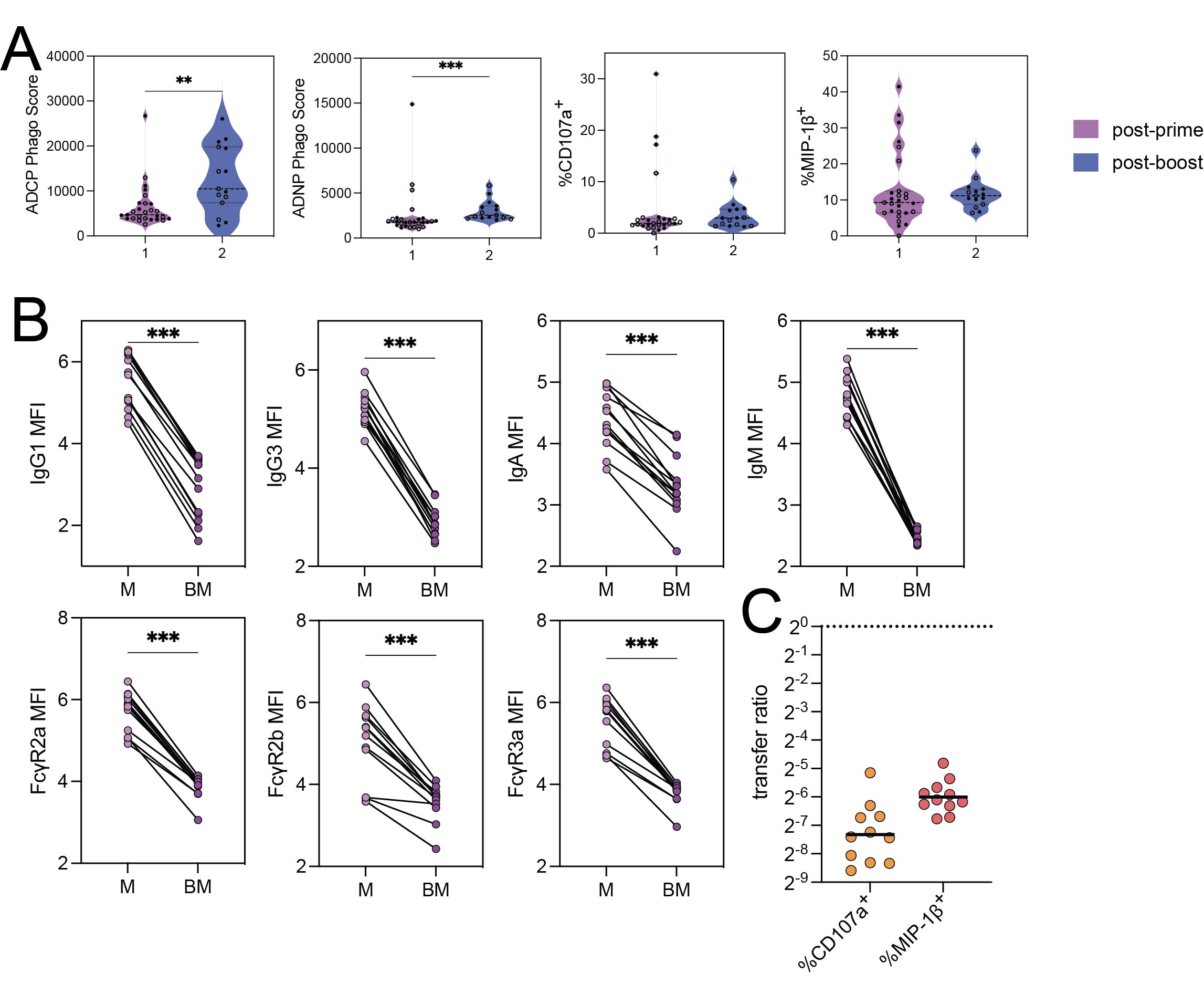

Supplement: FigureS4 — (A) The violin plots show the levels of ADCP, ADNP, and ADNKA (percent CD107a+ and MIP-1β+) activity in breastmilk at 3 to 4 weeks post-prime vaccination (n = 29, pink) and at 2 to 5.5 weeks post-boost vaccination (n = 15, blue). Data are presented as median±IQR. Significance was determined by a Mann-Whitney test. ** p < 0.01, *** p < 0.001. No asterisk indicates no significance was observed. (B) The dot plots show the IgG1, IgG3, IgA, IgM, FcγR2a-binding, FcγR2b-binding and FcγR3a-binding titers against SARS-CoV-2 spike post-boost vaccination in maternal serum (M) and breastmilk (BM). Lines connect matched maternal serum:breastmilk dyads (n = 13). Significance was determined by Wilcoxon-matched pairs signed rank test. *** p < 0.001. (C) The dot plots show the transfer ratio (breastmilk:serum) of ADNKA (CD107a and MIP-1b) activity at the post-boost timepoint. The dotted horizontal line indicates a transfer ratio of 1. The horizontal bars indicate the median of each group. Significance was determined by Wilcoxon-matched pairs signed rank test. No significant differences were observed. [file NIHMS1758174-supplement-FigureS4.jpg]

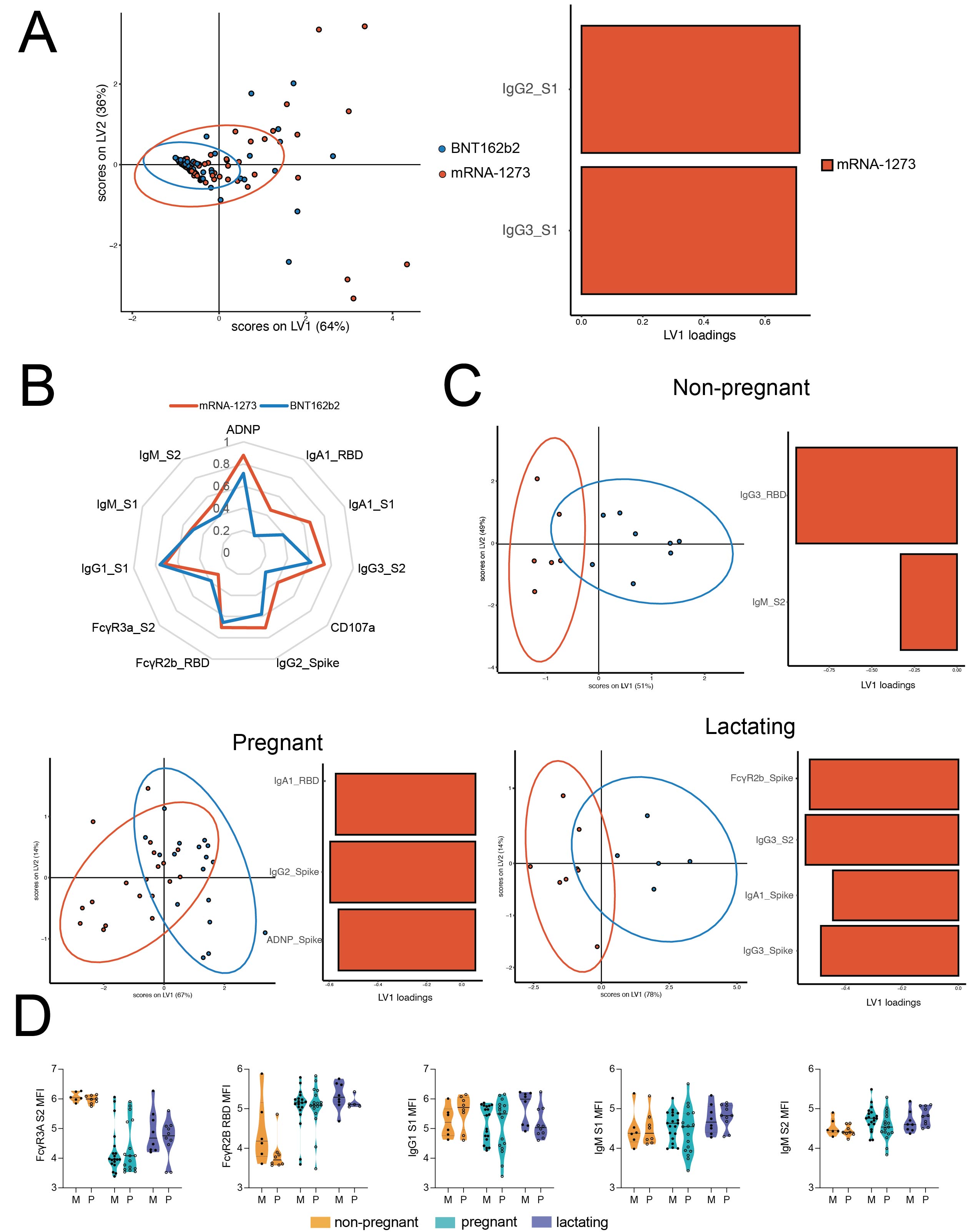

Supplement: FigureS5 — (A) A LASSO partial least squares-discriminant analysis (PLSDA) model was built on post-prime vaccination data from all groups. The dot plot (left) shows the scores for each sample, with each dot representing a sample. The ellipses represent the 95% confidence interval for each group. The bar plot (right) shows the loadings of each LASSO-selected feature, where the color marks the group enrichment. (B) The spider plot shows scaled values of each given antibody feature in each vaccine group. (C) A LASSO PLSDA model was built using V2 data from only non-pregnant, pregnant or lactating women. The dot plot shows the scores for each sample, with each dot representing a sample. The ellipses represent the 95% confidence interval for each group. The bar plot shows the loadings of each LASSO-selected feature, where the color marks the group enrichment. (D) The violin plots show differences in the top LASSO-selected features from Fig. 4A in non-pregnant (n = 8 for BNT162b2, n = 6 for mRNA-1273), pregnant (n = 17 for BNT162b2, n = 19 for mRNA-1273), and lactating (n = 5 for BNT162b2, n = 8 for mRNA-1273) women given either the mRNA-1273 (M, filled dots) or BNT162b2 (P, outline dots). Data are presented as median±IQR. Significance was only calculated between groups in the same time point and was determined by a one-way ANOVA followed by posthoc Šidák’s multiple comparison test. P-values were then corrected for multiple comparisons using the Bejamini-Hochberg procedure. No statistically significant differences were observed. [file NIHMS1758174-supplement-FigureS5.jpg]
